# Six Decades of Global Research on Bovine Babesiosis Vaccines: A Comprehensive Systematic Review and Meta-Analysis

**DOI:** 10.3390/pathogens15050500

**Published:** 2026-05-06

**Authors:** Uriel Mauricio Valdez-Espinoza, Chyntia Pérez-Almeida, Alma Cárdenas-Flores, Edwin Esaú Hernández-Arvizu, Juan Mosqueda

**Affiliations:** 1 Instituto Nacional de Investigaciones Forestales, Agrícolas y Pecuarias, Centro Nacional de Investigación Disciplinaria en Salud Animal e Inocuidad, Carretera Federal Cuernavaca, Cuautla No. 8534, Colonia Progreso, Jiutepec C.P. 62574, Mexico; 2 Immunology and Vaccines Research Laboratory, Natural Sciences College, Autonomous University of Querétaro, Querétaro 76230, Mexico; chyntiaq@gmail.com (C.P.-A.); alma.cardenas@uaq.mx (A.C.-F.); esau.hernandez@uaq.mx (E.E.H.-A.)

**Keywords:** Bovine babesiosis, cattle health, immunization, tick-borne disease, systematic review, vaccines

## Abstract

Bovine babesiosis is a tick-borne disease that poses significant economic losses to global cattle production, and diverse vaccine platforms have been developed to combat it. This work critically evaluates global research on bovine babesiosis vaccines, integrating historical milestones with recent advances in antigen discovery and immunization approaches and assessing their protective efficacy through meta-analysis. Using comprehensive database searches, we identified 413 publications on bovine babesiosis vaccines, of which 168 met the inclusion criteria, spanning from 1960 to August 2025. Analysis revealed that *B. bovis* dominated the research output, followed by *B. bigemina* and *B. divergens*. Five key methodological approaches emerged: field trials, controlled experiments, immunogenicity assessments, in vitro assays, and in silico antigen analyses, with a notable shift toward immunogenicity and computational studies post-2000. Frequently studied antigens included RAP-1, MSA-2c, AMA-1, 11C5, and 12D3 (*B. bovis*); RAP-1 and GP45 (*B. bigemina*); and Bd37 (*B. divergens*). Geographically, research was concentrated in the United States, Australia, Argentina, Mexico, and France, which were identified as the top contributors in that order, primarily focusing on live and recombinant vaccines, with minimal African participation despite high cattle populations. Bibliometric analysis showed increasing publication output, with leading journals such as *Veterinary Parasitology*, *Infection and Immunity*, *and Parasites & Vectors* as the top three. Thematic evolution highlighted a transition from live vaccines to recombinant and multi-epitope strategies, with increasing emphasis on conserved antigens and novel platforms. However, more field evaluations are required to determine whether these new technologies can achieve protective efficacy comparable to that of live vaccines. This work underscores the need for sustained investment, intersectoral collaboration, and validation using standardized and comparable metrics in field trials to translate laboratory innovations into effective, safe, and globally accessible vaccines against bovine babesiosis.

## 1. Introduction

Bovine babesiosis, also referred to as piroplasmosis, redwater, cattle fever, or tick fever, is a globally distributed, economically significant disease, primarily characterized by severe hemolytic anemia caused by infection of bovine erythrocytes with protozoan parasites of the genus *Babesia* [1,2]. The first description was provided by Victor Babes in 1888 [2] in Romania, who reported microorganisms within erythrocytes of cattle exhibiting hemoglobinuria, naming them *Haematococcus bovis*. In North America, Smith and Kilborne in 1893 [3] identified and named the etiological agent of Texas fever as *Pirosoma bigeminum*. These microorganisms were later renamed by Starcovici in 1893 [4] as *Babesia bovis* and *Babesia bigemina*, respectively [5]. In Europe, *Piroplasma divergens* was described in 1911 and was later reclassified as *Babesia divergens* [6,7]. In subsequent years, additional species were identified and classified, such as *Babesia ovata* in Japan [8], *B. major* in China [9], and recently, *B. naoakii* in Asia [10]. Currently, the genus *Babesia* is taxonomically classified within the phylum Apicomplexa, and several species are known to infect cattle worldwide, including *B. bovis*, *B. bigemina*, *B. divergens*, *B. major*, *B. ovata*, *B. occultans*, and *B. motasi* [4,5,6,7,8,9,10,11,12,13].

The geographic distribution of the etiological agents of bovine babesiosis is closely linked to the presence of their tick vectors, as well as the availability of competent hosts and environmental factors that sustain the climatic conditions necessary for maintaining the tick–pathogen–cattle cycle [14]. *Babesia bovis* and *Babesia bigemina* exhibit a global distribution due to the wide dispersal and adaptability of their main vectors, *Rhipicephalus microplus* and *R. annulatus*, which occur in Asia, Africa, the Americas, Australia, and southern Europe [15]. *Babesia divergens* is widely distributed in Europe, with reports also from North Africa [15,16,17,18]. *Babesia major*, *B. ovata*, *B. occultans*, and *B. motasi* have more restricted distributions in certain regions of Asia [18,19,20,21,22].

Bovine babesiosis has a global prevalence of 20–60%, with the highest rates in the Americas and Australia and a lower prevalence in Asia, and *B. bigemina* is reported as the most prevalent etiological agent [23]. Due to its worldwide distribution (see https://www.cabidigitallibrary.org/doi/10.1079/cabicompendium.91723, accessed on 4 May 2026), the economic losses associated with bovine babesiosis include mortality, abortions, reduced meat and milk production resulting from hemolytic anemia, as well as the costs of control measures and the decreased productive potential in endemic regions [23,24].

Given the significant impact of bovine babesiosis on global cattle productivity, decades of research have prioritized disease control, with vaccine development emerging as a leading strategy [4,25,26,27,28,29]. While some of these vaccines have been used experimentally, others are commercially available. Florin-Christensen et al. [30] compiled a list of vaccines against *Babesia bovis* and *B. bigemina* used in countries including Argentina, Australia, Brazil, Colombia, Israel, Malawi, Mexico, South Africa, and Uruguay.

These vaccines have been a cornerstone of herd health programs, particularly in high-demand regions like Australia and Argentina [31,32]. In some instances, vaccines have been imported for experimental use in non-producing countries [33]. In Argentina, for instance, Spath et al. [32] reported that 70% of vaccine applications target breeding cattle, primarily those moved from tick-free regions to endemic or enzootic unstable areas.

In recent years, the detection of bovine babesiosis agents in atypical geographic regions [34,35,36], combined with environmental changes that promote the geographic expansion of tick vectors [37,38,39], suggests that the demand for vaccines against these pathogens will persist or even increase. Consequently, this area is expected to remain an important focus of scientific investigation worldwide [40,41,42,43,44].

Globally, significant research has been undertaken to identify and evaluate methodologies for developing bovine babesiosis vaccines [28,45,46,47,48,49,50]. A major output of this field is the scientific literature generated over decades, available in health science databases such as PubMed^®^ from the National Center for Biotechnology Information (NCBI) and Web of Science^®^. Systematic reviews have been recognized as an effective approach to assessing the body of literature across different research areas, addressing theoretical and methodological questions, and identifying patterns and trends [51]. Furthermore, such reviews are valuable tools for analyzing the evolution of research fields [52,53,54].

In the context of babesiosis, recent systematic reviews have synthesized studies focused on infection rates and scientific research output [55,56]. Specifically for bovine babesiosis, Jacob et al. [23] reported the global prevalence of the disease through a systematic review and meta-analysis, while more recently, Santos et al. [57] discussed vaccine development against *Babesia bovis* over the past decade. However, to date, no global systematic review has comprehensively addressed the development of vaccines targeting all etiological agents of bovine babesiosis.

This systematic review aims to provide the first comprehensive, worldwide synthesis of the historical and current development of vaccines against bovine babesiosis. In addition, it performs an integrated bibliometric and scientometric assessment to examine research trends, identify critical knowledge gaps, and propose future directions for advancing this field. Furthermore, it presents the first integrated evaluation of vaccine protective efficacy based on studies conducted over decades of research and across the different technological approaches developed.

## 2. Materials and Methods

This systematic review followed the guidelines of the Preferred Reporting Items for Systematic Reviews and Meta-Analyses (PRISMA 2020) statement [58]. We provide the PRISMA checklist in Appendix A. The review protocol was registered in the Open Science Framework and is available at https://doi.org/10.17605/OSF.IO/KU5QB (Registered on 23 September 2025).

### 2.1. Literature Search Strategy

To retrieve scientific information related to the topic, we conducted the first literature search in the PubMed^®^ database from the National Center for Biotechnology Information (NCBI) and in Web of Science^®^ on 13 December 2024. To account for the advances of the year 2025, the search was updated on August 13. The search string used the following keywords and Boolean operators: ((Bovine babesiosis OR Cattle babesiosis) AND (vaccine OR recombinant vaccine OR subunit vaccine) AND (proteins OR antigens OR peptides)).

### 2.2. Inclusion and Exclusion Criteria

We included all the publications that were available up to the search date. Articles were initially screened by title, selecting only those that made specific reference to bovine babesiosis. Publications referring to bovine anaplasmosis or theileriosis were excluded. Review articles, systematic reviews, meta-analyses, opinion pieces, and commentaries were also excluded, retaining only original research articles. Duplicate records across databases were removed.

Abstracts and methodologies of pre-selected studies were then individually reviewed. We included only those addressing one or more of the following topics: identification of vaccine proteins or antigens, recombinant antigens, controlled immunization trials, cattle immunization under field conditions, in vitro assays, immune response evaluation, in silico antigen analysis, development of live or live-attenuated vaccines, and studies on the genetic diversity of vaccine strains of the parasites. Studies focusing exclusively on diagnostic methods for bovine babesiosis were excluded. The final set of selected publications (168 studies) was used to perform the analyses corresponding to the systematic review, as described in the following section.

Selected studies referred to the etiological agents of bovine babesiosis: *Babesia bovis*, *B. bigemina*, *B. divergens*, and *B. major*.

### 2.3. Data Extraction and Bibliometric, Scientometric, and Methodological Analysis

From the selected publications, we extracted the following information: title and year of publication, *Babesia* species addressed in the study, type of vaccine evaluated, type and number of antigens used, antigen origin, type of experiment conducted, and country where the study was performed. All data were organized and stored in a .csv file (Supplementary Material S1).

This .csv database was used to perform descriptive analyses and graphical representations in the R programming environment [59], employing the packages tidyverse [60], viridis [61], and ggplot2 [62]. Additionally, ArcMap 1.8© software was used for spatial visualization of the countries where the research was conducted. Figures were processed in Inkscape^®^ to enhance their graphical presentation and adapt them to the publication format.

For the bibliometric and scientometric analyses, BibTeX files were exported from Web of Science© and PubMed©. These files contained all metadata of the selected bibliography (Supplementary Material S2) and served as the basis for the analyses.

We used the Bibliometrix package [63] to perform bibliometric and scientometric analyses of the field. Multiple dimensions were analyzed, including sources of information, authorship, scientific production, social structure (collaboration among authors and institutions), and intellectual structure (historiographic analysis). We also identified trending topics and titles, keyword co-occurrence networks (from titles, abstracts, and keywords), and patterns of international collaboration.

Finally, to identify and represent the key topics and concepts in this field and their temporal evolution, we developed thematic co-occurrence maps showing centrality and density values of the selected items, which indicate their importance and level of development [48,49]. Thematic evolution maps were created for the periods 1967–1991, 1992–2007, 2008–2018, 2019–2024, and 2025. For their creation, we selected Keyword Plus, authors’ keywords, titles, and abstracts from the metadata. Some studies, particularly those from the early years of research, could not be included due to incomplete metadata.

### 2.4. Meta-Analysis of Vaccine Protective Efficacy Across Reported Studies

From the 168 publications identified in the systematic review, we selected 85 studies for this analysis. These studies were classified as evaluations using controlled experiments and field experiments in cattle models, in which direct immunization followed by challenge was performed. Data extraction was carried out through independent manual review by a team member who did not participate in the previous stages of the study, in order to minimize bias. Qualitative and quantitative metadata were entered into a spreadsheet, including experimental design variables and outcomes (Supplementary Material S6). Methodological assessment was performed according to the criteria described by Vesterinen et al. [64]. Analyses were performed using R software (utilizing the metafor package) [65].

For the protection against mortality outcome, effect sizes were estimated as odds ratios (OR). Non-informative studies with double-zero events (0% or 100%) were excluded. Cells with single-zero events were addressed using the default correction implemented in metafor. Records with logical inconsistencies or incomplete data were removed. Pooled effects were estimated using random-effects models with a restricted maximum likelihood (REML) estimation, estimating both I^2^ and between-study variance (τ^2^).

For continuous outcomes (temperature and hematocrit after the post-vaccination disease challenge), the standardized mean difference (SMD) was calculated using Hedges’ g, which incorporates a correction for bias associated with small sample sizes. Only studies with means, standard deviations, and sample sizes available for both groups were included. For hematocrit, when the outcome was explicitly reported as a “reduction”, the direction of the effect size was inverted to ensure biological coherence.

The significance of the overall effect was assessed using the Z-statistic. Leave-one-out sensitivity analyses were performed to evaluate the individual influence of each study on mortality. Potential sources of heterogeneity were explored using meta-regression with categorical moderators, obtaining independent estimates per category for vaccine type, experiment type, group size, and efficacy according to the *Babesia* species. Statistical significance was considered at *p* < 0.05. ORs < 1 were interpreted as a protective effect, and the magnitude of the SMD was interpreted according to conventional thresholds.

Different meta-analytical approaches were conducted, and the number of studies included in each analysis depended on the availability of the complete data for the selected outcomes (mortality protection, temperature, and hematocrit). Many publications were excluded or used only for qualitative synthesis due to missing methodological information or inconsistent reporting. Articles containing multiple independent trials were treated as separate analytical units; although 74 studies met some inclusion criteria, only those with complete information were included in each outcome-specific meta-analysis.

To explore sources of variation in vaccine efficacy, subgroup analyses were performed according to vaccine technology, type of experiment (controlled and field), and *Babesia* species. When possible, continuous outcomes were analyzed separately for controlled and field studies, and a global analysis combining all the studies reporting temperature and hematocrit was also conducted to strengthen overall inference.

To ensure the robustness of the meta-analysis results, publication bias and small-study effects were assessed for all outcomes (mortality, body temperature, and hematocrit). Funnel plots were visually inspected using contour-enhanced funnel plots, which help distinguish asymmetry potentially caused by publication bias from other sources of heterogeneity by displaying statistical significance regions (*p* > 0.10, 0.05 < *p* ≤ 0.10, 0.01 < *p* ≤ 0.05, and *p* ≤ 0.01). To address the overlapping points caused by identical effect sizes and standard errors, semi-transparent plotting was applied to improve the visualization of the study density.

Statistical asymmetry was evaluated using Egger’s regression test and Begg’s rank correlation test. In addition, the Duval and Tweedie trim-and-fill method was applied to estimate the number of potentially missing studies and to compute an adjusted pooled effect size. All analyses were conducted using the metafor package in R [65]. A *p*-value < 0.05 was considered indicative of significant publication bias.

## 3. Results

### 3.1. Scientific Literature

**Figure 1:**
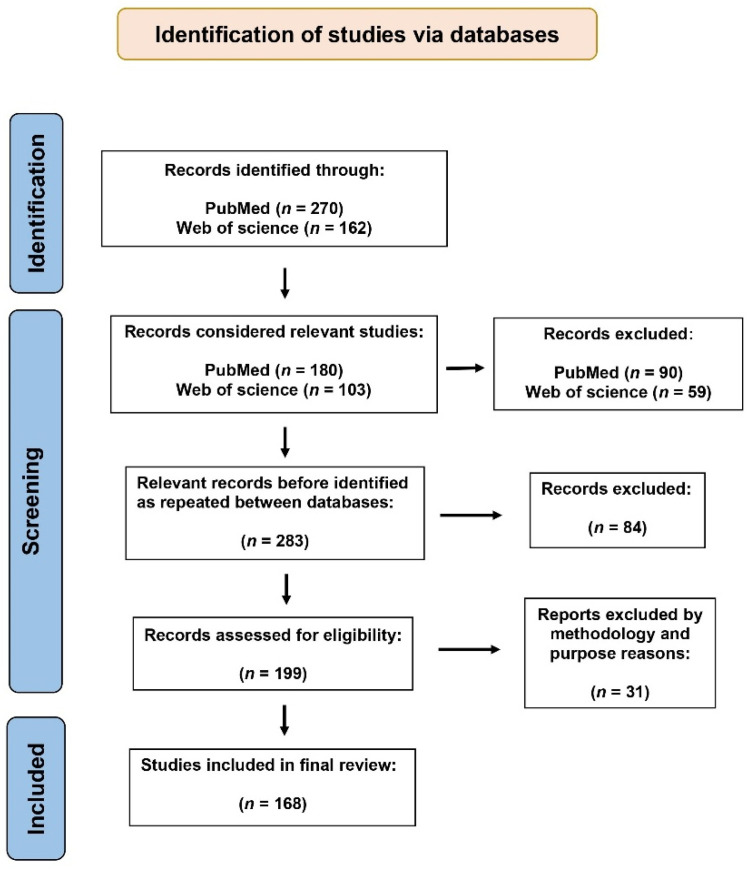
A PRISMA flow diagram illustrating the database search process and the selection of studies included in this study.

The database search yielded a total of 432 publications. Of these, only 283 specifically referred to vaccines against bovine babesiosis, and after applying the eligibility criteria, 168 publications were selected for the global synthesis (Figure 1). The selected publications are listed in Supplementary Material S1. The final set spans from September 1960 to August 2025, representing more than six decades of bibliographic information.

### 3.2. Development of Vaccines Against Bovine Babesiosis

**Figure 2:**
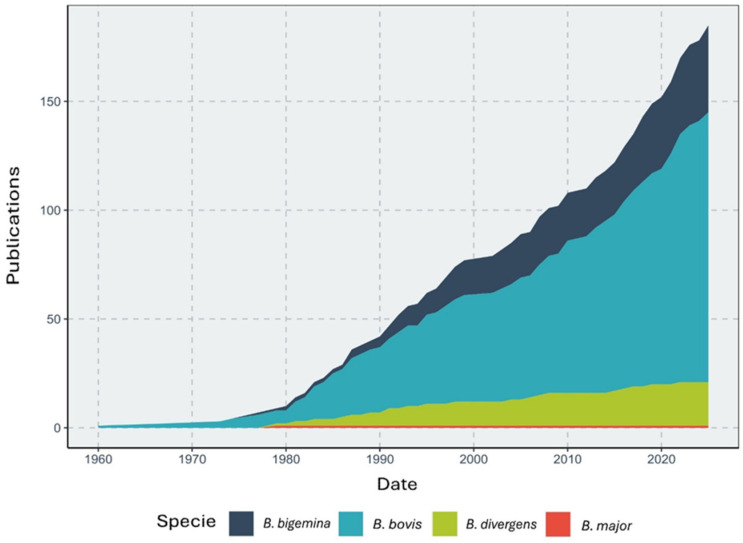
The *Babesia* species considered in vaccine development over time, based on the publications selected in this study. The graph was adapted from Lippi et al. (2021) [66].

*Babesia bovis* is the etiological agent with the largest number of publications in this field, followed by *B. bigemina* and *B. divergens* (Figure 2). *B. major* has received minimal attention in vaccine development research (Figure 2). Some studies evaluated vaccines against both *B. bovis* and *B. bigemina* simultaneously (see Supplementary Material S3). The number of publications per species shows an overall upward trend over time.

**Figure 3:**
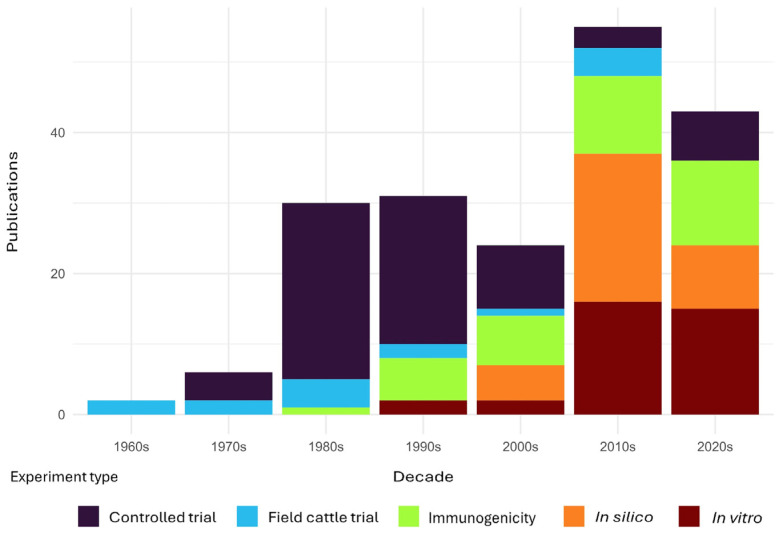
Methodologies used in the development and evaluation of vaccines against bovine babesiosis, grouped by decade.

Five main methodological approaches were identified in the development and evaluation of bovine babesiosis vaccines: (i) field protection trials, (ii) controlled or stable experiments, (iii) immunogenicity evaluation, (iv) in vitro assays, and (v) in silico antigen analyses (Figure 3). Decadal analysis revealed that until the 1990s, field trials and controlled experiments were the predominant methodologies (Figure 3). From that period onward, there has been a marked shift toward methodologies focused on the immunogenicity evaluation of vaccine candidates, along with in vitro and in silico assays (Figure 3). Currently, these approaches are the most widely employed (Figure 3), although such studies have generally not progressed to challenge trials in cattle. A consistent pattern of immunogenicity evaluation of antigens has been observed since the 2000s.

**Figure 4:**
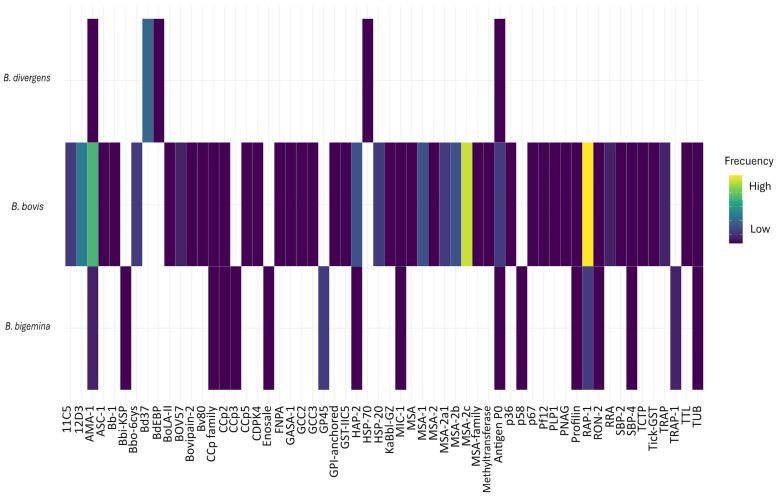
A heatmap representing the frequency of evaluation of antigens considered in the development of vaccines against bovine babesiosis, according to *Babesia* species.

Since the 1980s and early 1990s, research efforts have targeted the identification and evaluation of antigens as potential vaccine candidates against bovine babesiosis. Numerous antigens have been investigated in various studies, with the greatest diversity assessed in *B. bovis*, followed by *B. bigemina*, and to a lesser extent, *B. divergens* (Figure 4). The most frequently evaluated antigens include RAP-1, MSA-2c, AMA-1, 11C5, and 12D3 in *B. bovis*; RAP-1 and GP45 in *B. bigemina*; and Bd37 in *B. divergens* (Figure 4). Only AMA-1 and P0 have been evaluated in all three *Babesia* species, while CCp family antigens, HAP-2, MIC-1, profilin, RAP-1, RON2, and SBP4 have been studied in both *B. bovis* and *B. bigemina* (Figure 4).

**Figure 5:**
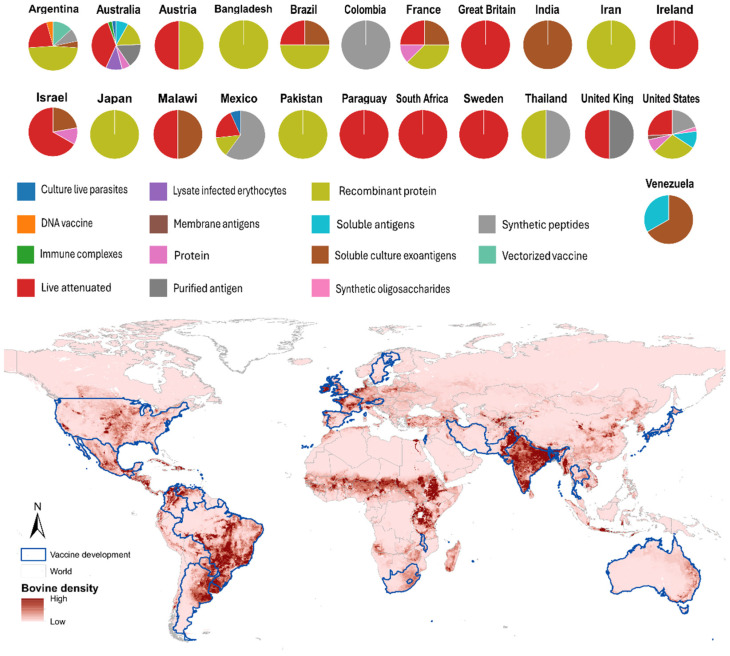
The countries conducting research on vaccines against bovine babesiosis. The proportion of research effort by vaccine model is shown per country. Blue-bordered polygons highlight the countries where this work has been carried out. Cattle density layer source: https://www.fao.org/livestock-systems/global-distributions/cattle/en/ (accessed on 4 May 2026).

Globally, several countries have developed vaccine-related methodologies against bovine babesiosis (Figure 5). In some countries, multiple vaccine models have been evaluated, whereas others have focused on one or a few specific types (Figure 5). Australia, the United States, and Argentina have conducted the largest number of studies in vaccine design and evaluation, most frequently testing live vaccines, followed by recombinant protein-based and synthetic peptide-based models, in which Mexico has made the greatest progress (Figure 5). A spatial analysis of this research effort reveals a strong correlation with cattle demographics, showing that countries with abundant cattle populations are generally those involved in this research. However, a notable and significant lack of participation is observed among African nations, despite their substantial cattle populations, as shown in Figure 5. This disparity highlights a critical gap in the global research landscape, as many African regions are endemic for babesiosis and stand to benefit greatly from vaccine development.

The limited involvement from Africa can be attributed to a complex interplay of factors, including but not restricted to: limited veterinary and research infrastructure, significant funding constraints, challenges in navigating international biosafety regulations for live vaccines, and barriers to technology transfer that hinder the local development and evaluation of modern vaccine platforms like recombinant and peptide-based models. This confluence of challenges has resulted in a research geography that does not fully align with the disease’s economic and agricultural impact.

### 3.3. Bibliometric and Scientometric Analysis

A total of 164 publications in the BibTeX format were used for analysis. Annual scientific output in this field has fluctuated over time but shows a general upward trend (Supplementary Material S5; Figure S1). Publications were distributed across 54 scientific journals, with *Veterinary Parasitology*, *Infection and Immunity*, *Parasites & Vectors*, *International Journal for Parasitology*, and *Vaccine* as the main sources. Among these, *Infection and Immunity* and *Veterinary Parasitology* were the most frequently cited (see details in Supplementary Material S5; Figures S2–S4).

Suarez C.E. was identified as the most prolific author in this field. A temporal authorship pattern was observed: prior to 2000, the leading authors were Wright I.G., Goodger B.V., and Waltisbuhl D.J., whereas from 2000 onwards, Suarez C.E., along with other emerging authors, gained prominence. The top ten authors are listed in Supplementary Material S5; Figures S5 and S6. The leading authors are affiliated with institutions from the United States, Australia, Argentina, Mexico, France, Japan, Brazil, Ireland, and South Africa, in that order. Participation from these countries has shown an upward trend over time. Additional details on author affiliations and institutional participation are provided in Supplementary Material S5; Figures S7–S12.

**Figure 6:**
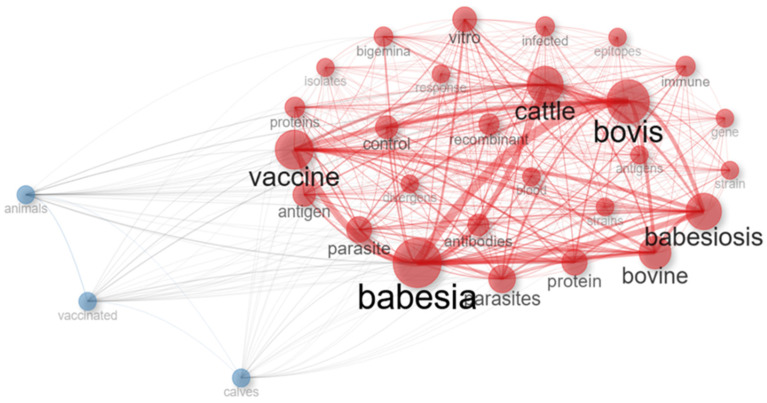
Co-occurrence network of the most frequent terms in publications on vaccine development against bovine babesiosis. Node size indicates term frequency, while link strength reflects relationship intensity.

Conceptual analysis indicates that this field is strongly centered on vaccine design and evaluation against *B. bovis* (Figure 6). Relevant keywords and co-occurrence networks show that concepts related to *Plasmodium falciparum*, immunology, antigens, recombinant techniques, vaccination, and antibodies are central and densely interconnected within this research domain. Nonetheless, areas with lower connectivity were identified, potentially representing emerging niches or future application-oriented directions (Figure 6; Supplementary Material S5; Figures S13–S20).

**Figure 7:**
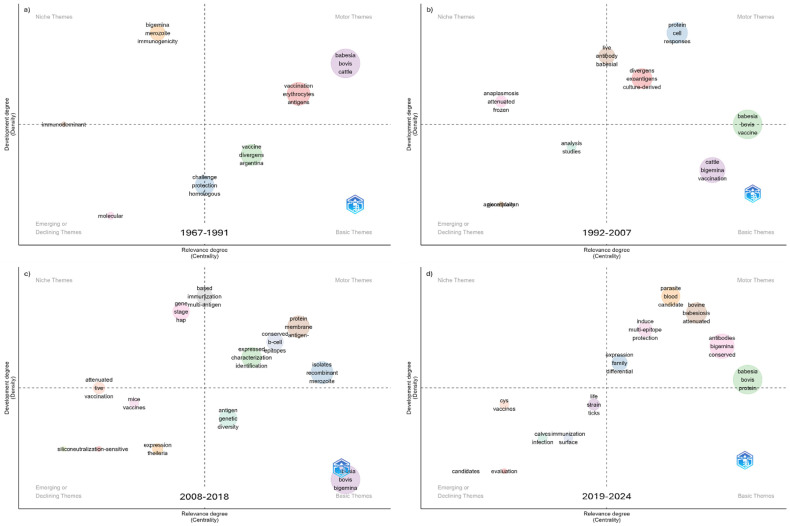
Conceptual evolution in the development of vaccines against bovine babesiosis, represented through thematic maps. (**a**) 1967–1991, (**b**) 1992–2007, (**c**) 2008–2018, and (**d**) 2019–2024.

Thematic evolution analysis revealed that between 1967 and 1991, topics related to *B. bovis*, vaccination, antigens, and erythrocytes were well-developed. Research on *B. divergens* vaccines was an important theme, whereas *B. bigemina* was addressed more sporadically. Immunogenicity appeared as an incipient line of research, as did molecular techniques (Figure 7a).

Between 1992 and 2007, themes related to parasite culture-based methodologies, live vaccines, proteins, and exoantigens in vaccine development became well-established. During this stage, *B. bigemina* gained greater relevance compared to the previous period. Topics related to attenuated and frozen vaccines were explored only peripherally, while methodologies specifically targeting the Apicomplexa phylum, to which the etiological agents belong, began to emerge (Figure 7b).

From 2008 to 2018, research on antigens, recombinant techniques, and conserved B-cell epitopes became the most developed and central themes. In contrast, the use of live vaccines showed a marked decline in importance. Identification of genes associated with vectors was explored only peripherally, while the genetic diversity of antigens was recognized as important but underdeveloped (Figure 7c).

In the period 2019–2024, research on vaccine candidates, conserved multi-epitope vaccines, and attenuated vaccines continues to drive the field, whereas topics related to immunization are positioned as either declining or emerging themes (Figure 7d).

Finally, in the most recent year (2025), a similar research trend was observed in multi-epitope vaccine studies; however, in this case, *B. bigemina* has been more extensively explored (Supplementary Material S5; Figures S21 and S22).

### 3.4. Protective Efficacy of Vaccines Across Studies

A considerable number of studies were excluded due to the absence of methodological information or inconsistencies in reporting format. Overall, 74 studies met the inclusion criteria; however, complete information was not available for all selected outcomes (mortality, body temperature, and hematocrit). Consequently, the number of studies included varied across analyses and is indicated in each result section. Some articles included multiple independent trials, which were treated as separate analytical units. Accordingly, part of the evidence was used for qualitative synthesis, whereas studies with sufficient data were included in quantitative meta-analysis. *Babesia divergens* was excluded from the mortality analysis due to the insufficient number of studies and available information.

A chronological trend was observed in the dose used, with initial volumes of 10–12 mL progressively decreasing to 1–2 mL in more recent studies. The subcutaneous route of administration was the most commonly used (>50% of studies). The Holstein breed was the most frequently used, followed by Hereford and Angus.

#### 3.4.1. Mortality Protection

**Figure 8:**
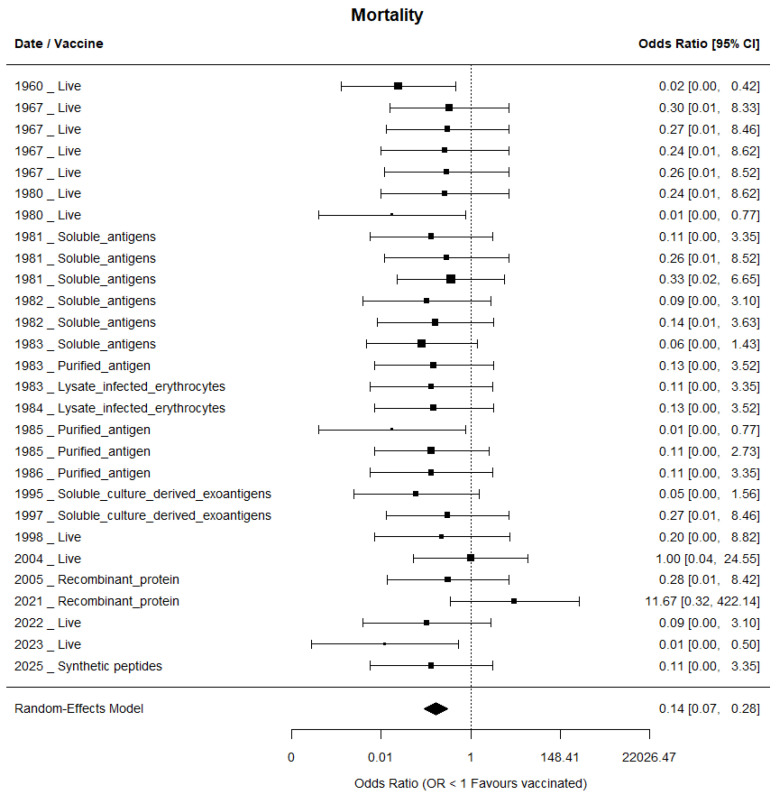
A forest plot showing the comparison of mortality between vaccinated and non-vaccinated groups. Individual studies are identified by year and vaccine type. The black squares represent the odds ratio (OR) for each study, with the square size proportional to the inverse variance weight. The horizontal lines indicate 95% confidence intervals. The vertical dashed line represents the null effect (OR = 1). The black diamond at the bottom represents the pooled OR estimated using a random-effects model with restricted maximum likelihood (REML), with its width corresponding to the 95% confidence interval. Odds ratios < 1 indicate a protective effect of vaccination.

The meta-analysis of 29 studies showed a significant protective effect of vaccination on mortality (overall OR = 0.14). No heterogeneity was detected between studies (I^2^ = 0%), suggesting consistency in the magnitude of the observed effect. The overall effect was highly significant (Z = −5.84; *p* < 0.001) (Figure 8). Leave-one-out sensitivity analysis confirmed the stability of the pooled estimate, indicating that the protective effect was not disproportionately influenced by any individual study (Supplementary Material S7; Figure S1).

#### 3.4.2. Analysis by Vaccine Technology

The number of studies included by vaccine technology was as follows: 11 live vaccines, six soluble antigens, four purified antigens, three lysates of infected erythrocytes, two soluble culture-derived exoantigens, two recombinant proteins, and one synthetic peptide. Subgroup analysis revealed differences in the magnitude of the effect according to the technology used. Platforms based on purified antigens presented the lowest OR (0.08; 95% CI: 0.01–0.44), followed by live attenuated parasites (OR ≈ 0.11–0.14) and soluble antigens (OR: 0.14; 95% CI: 0.04–0.54), all with confidence intervals that did not cross unity, indicating a statistically significant protective effect. In contrast, technologies based on synthetic peptides (OR: 0.11; 95% CI: 0.00–3.35) and recombinant proteins (OR: 1.64; 95% CI: 0.14–19.29) presented wide confidence intervals that included unity, which precludes concluding a significant protective effect in these cases (Supplementary Material S7; Figure S2).

#### 3.4.3. Type of Experiment

The studies comprised 23 controlled experimental trials and six field cattle studies. The mixed-effects model indicated that the type of experiment significantly explained part of the variability in vaccine efficacy (QM = 34.44; *p* < 0.0001), with no detectable residual heterogeneity (I^2^ = 0%). Both controlled experimental studies (OR ≈ 0.13) and field studies (OR ≈ 0.21) showed significant protective effects, although the magnitude of the effect was greater under the controlled experimental conditions. This difference could be influenced by the higher number of controlled studies compared to the field studies.

#### 3.4.4. *Babesia* Species

In this evaluation, five studies corresponded to *B*. *bigemina* and 24 to *B*. *bovis*. Parasite species significantly explained the variation in vaccine efficacy (QM = 35.57; *p* < 0.0001), with no detectable residual heterogeneity (I^2^ = 0%). Significant protective effects were observed against both *Babesia bovis* (OR ≈ 0.13) and *Babesia bigemina* (OR ≈ 0.13), with similar effect magnitudes existing between both species.

#### 3.4.5. Body Temperature and Hematocrit in Vaccine Challenge

*Controlled Experiments*

- *Body Temperature*

The meta-analysis of 16 studies showed a significant protective effect of vaccination on the febrile response (overall SMD = −2.45; 95% CI: −3.03 to −1.86). The random-effects model confirmed a significant reduction in temperature in vaccinated cattle compared to controls (Z = −8.16; *p* < 0.0001). Moderate heterogeneity was observed (I^2^ = 52.26%), indicating variability between the experimental models and the challenge conditions used in the different studies (Supplementary Material S7; Figure S3).

*Hematocrit*

The meta-analysis of 29 studies showed that a significant protective effect on hematocrit was observed (overall SMD = 2.83; 95% CI: 1.81–3.86), evidencing greater erythrocyte preservation in vaccinated cattle (Z = 5.41; *p* < 0.001). Heterogeneity was extremely high (I^2^ = 90.71%), indicating a marked variability between studies. However, the direction of the effect remained consistently in favor of vaccination, particularly in studies with live vaccines, soluble antigens, in vitro cultures, and the RAP-1 recombinant antigen (Supplementary Material S7; Figure S4).

- *Field Experiments*

*Body temperature*

The meta-analysis of eight studies indicated a significant protective effect of vaccination (overall SMD = −1.84; 95% CI: −3.06 to −0.63). The random-effects model confirmed a significant reduction in the febrile response in immunized cattle (Z = −2.98; *p* = 0.0029). High heterogeneity was detected (I^2^ = 82.34%), reflecting the variability inherent to real production conditions, differences in management, natural exposure, and technological diversity (Supplementary Material S7; Figure S5).

*Hematocrit*

It was not possible to perform a meta-analysis for hematocrit in field studies due to the insufficient number of studies available with the required information.

- *Subunit Vaccines in Controlled Experiments*

*Body Temperature*

In the meta-analysis of five studies evaluating recombinant protein-based vaccines, no statistically significant protective effect was observed (overall SMD = −0.14; 95% CI: −0.74 to 0.47; Z = −0.45; *p* = 0.655). No heterogeneity was detected between the studies (I^2^ = 0%), indicating consistency in the absence of effect across the five studies evaluated (1997–2024) (Supplementary Material S7; Figure S6).

*Hematocrit*

Similarly, the meta-analysis of eight studies found a significant effect on hematocrit (overall SMD = 0.11; 95% CI: −0.32 to 0.55; Z = 0.51; *p* = 0.612). Heterogeneity was null (I^2^ = 0%), suggesting consistently neutral results in the eight studies analyzed (1995–2024) (Supplementary Material S7; Figure S7).

#### 3.4.6. Global Analysis of Body Temperature and Hematocrit Values

This analysis included 32 studies reporting body temperature and 45 studies reporting hematocrit. It was conducted to provide additional support and enhance the statistical robustness of the findings.

*Body Temperature*

The global meta-analysis showed a significant protective effect of vaccination (overall SMD = −1.47; 95% CI: −2.20 to −0.75; Z = −3.98; *p* < 0.0001). However, very high heterogeneity was observed (I^2^ = 86.92%), indicating that the magnitude of the effect varies considerably between studies. This variability suggests that temperature values depend on factors such as vaccine technology, the experimental model, and challenge conditions (Supplementary Material S7; Figure S8).

*Hematocrit*

A significant protective effect on hematocrit was observed (overall SMD = 1.56; 95% CI: 0.64–2.48; Z = 3.33; *p* = 0.0008). Heterogeneity was extremely high (I^2^ = 93.59%), reflecting the substantial differences between vaccine platforms and experimental conditions. In general, studies using live vaccines showed larger effect sizes, whereas recombinant technologies tended to yield effects closer to neutrality (Supplementary Material S7; Figure S9).

### 3.5. Risk of Bias Assessment

**Figure 9:**
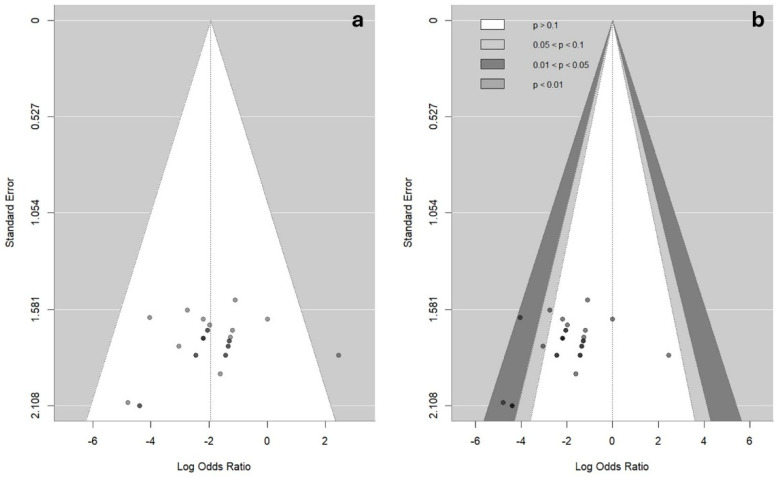
An evaluation of publication bias in bovine babesiosis vaccine trials for the mortality outcome. (**a**) A standard funnel plot with transparency to highlight overlapping studies with identical effect sizes and standard errors. (**b**) A contour-enhanced funnel plot showing that most trials fall within statistically significant regions (*p* < 0.05), with several located in the *p* < 0.01 region. The visual symmetry observed in both panels, supported by Egger’s test (*p* = 0.205), Begg’s test (*p* = 0.146), and the absence of imputed studies in the trim-and-fill procedure (k = 0), suggests a low risk of publication bias.

The risk of bias analysis based on funnel plot asymmetry for the mortality protection outcome (Figure 9) showed no significant evidence of publication bias. Egger’s regression test (z = −1.26, *p* = 0.205) and Begg’s rank correlation test (*p* = 0.146) indicated no statistically significant asymmetry. Furthermore, the trim-and-fill procedure did not impute missing studies (k = 0), supporting the robustness of the pooled estimate (Log OR = −1.94, 95% CI: −2.59 to −1.28; *p* < 0.0001), with no detected heterogeneity (I^2^ = 0.0%).

The assessment of publication bias for body temperature and hematocrit outcomes showed mixed evidence of funnel plot asymmetry (Supplementary Material S7; Figure S10). Egger’s regression test was not significant for either temperature (z = −0.74, *p* = 0.458) or hematocrit (z = 0.61, *p* = 0.536). However, Begg’s rank correlation test indicated significant asymmetry for temperature (Kendall’s τ = −0.3710, *p* = 0.0025), whereas hematocrit showed no significant asymmetry (Kendall’s τ = 0.1919, *p* = 0.0642). The trim-and-fill procedure suggested minor adjustments, indicating that potential small-study effects cannot be fully excluded.

## 4. Discussion

After decades of research on the biological aspects and control of bovine babesiosis, this disease continues to pose a serious challenge to the cattle industry [67,68]. Recently, Santos et al. [57] attempted to address the state of the art in bovine babesiosis vaccines; however, their review presented limitations in geographic coverage, methodological approaches, and temporal scope. Similarly, Alzan et al. [69] addressed this topic in a general manner, with bibliographic constraints. In this context, our findings provide an integrated, global, and historical contribution to the state of the art of vaccine development against bovine babesiosis worldwide.

*Babesia bovis* has become the primary focus of vaccine research and development, despite *B. bigemina* having an equal or even broader geographic distribution based on its vectors [15]. Our results in Figure 2 support this pattern. Similarly, the studies included in the meta-analysis show a greater proportion of research focused on *B*. *bovis* compared with *B*. *bigemina*. This bias toward *B. bovis* may be driven by its greater pathogenicity, virulence, and the more substantial economic losses it causes [70]. Although *B. divergens* and *B. major* play important epidemiological roles in Europe and parts of Asia, respectively, their more localized distributions are reflected in a lower number of publications compared with *B. bovis*. Nonetheless, in the case of *B. divergens*, the research output is comparable to that of *B. bigemina*, indicating notable progress for this etiological agent.

The temporal representation of methodologies in this field shows a significant shift beginning in the 2000s. Since then, genetic engineering-based strategies, driven by advances in omics sciences and their application to these pathogens, have gained prominence. This trend has been facilitated by the identification of genes and the sequencing of genomes of multiple *Babesia* species worldwide, enabling a greater understanding of their molecular biology, invasion mechanisms, and the identification of candidate genes for vaccine development [71,72,73,74].

The temporal pattern indicates that the most frequently employed approaches to date are in silico analyses, immunogenicity evaluation, and in vitro assays, consistent with strategies proposed by several research groups worldwide [29,69,75,76]. This approach aligns with the concept of reverse vaccinology, which involves: (i) in silico identification of candidate genes in the parasite genome; (ii) chemical synthesis and/or recombinant expression of these candidates to evaluate their immunogenicity through immunization trials; and (iii) assessment of their neutralizing capacity via in vitro assays [69,75,76].

A wide diversity of antigens from bovine babesiosis agents has been described (Figure 4) and evaluated using the methodologies outlined in this work. A meta-analysis of the results obtained with the most extensively studied antigens and vaccine formulations would be valuable to draw stronger inferences regarding their protective capacity [77], as recently suggested [76]. This issue is further discussed at the end of the section.

The United States leads in the number of publications in this field (Supplementary Material S4); however, our results indicate that Australia has made the greatest effort in developing methodologies and evaluating vaccine models against bovine babesiosis (Figure 5) and is recognized as a pioneer in this area (Supplementary Material S4) [78,79,80,81]. From a continental perspective, the Americas lead research output in this field, with the United States, Argentina, and Mexico ranking among the top five global contributors (Supplementary Material S4). In Latin America, countries such as Mexico, Brazil, and Argentina have made significant contributions, including the evaluation of live-attenuated vaccines [28,32], the use of in vitro parasite cultures [42], and the development of recombinant technology-based vaccine models [49,82,83]. In Europe, regarding *B. divergens*, France stands out as the leading country in this area (Figure 5). The countries involved in this research are generally located in regions with high cattle density; however, specific challenges, such as issues related to cattle vaccination and immunization, have emerged as areas requiring increased attention (Figure 5).

Our conceptual and temporal evolution analysis provides a framework to identify key aspects for both the current status and future directions of this field. The co-occurrence network (Figure 6) shows a strong connection between topics related to recombinant and/or subunit vaccine development. However, issues such as vaccination and immunization challenges in cattle emerge as areas requiring greater attention. This observation aligns with patterns identified in our results, where such topics appear as declining or emerging themes that may require further consolidation.

We documented the conceptual evolution of this field, highlighting trends from the emergence of molecular methodologies to their current dominance, alongside the decline of whole-parasite or live-attenuated vaccines. Between 1992 and 2007, studies focusing on the Apicomplexa phylum began to emerge, suggesting a conceptual expansion toward phylogenetic and molecular approaches to bovine babesiosis pathogens—a trend that became consolidated during 2008–2018. Bioinformatics tools have played a key role in this process. *Plasmodium falciparum* and other Apicomplexa have become relevant models for vaccine design against bovine babesiosis [82,83,84,85]. An example is the study of AMA-1 and RAP-1 proteins, which are located in specialized organelles and are essential for host cell invasion; these proteins were first identified in *Plasmodium*. Through such tools, *Babesia bigemina* has been incorporated into this research domain, enabling the identification of various vaccine candidate antigens, some of which are shared with *B. bovis* and *B. divergens* [72,84,85].

Antigen genetic diversity has emerged as a topic requiring greater attention—a conclusion supported not only by recent reports [57] but also by this scientometric analysis. This issue is closely linked to vaccine efficacy failures [86]. Santos et al. [57] emphasized the need to evaluate vaccines in different cattle populations, as in previous studies such as Brizuela et al. [33], where a vaccine developed in Australia was tested in Paraguayan herds with positive protection outcomes [33,87]. An even more relevant step would be to adopt a strategic selection of cattle populations based on their ecological environments, enabling more comprehensive inferences.

The availability of genetic sequences in databases, combined with environmental analyses, can strengthen recombinant vaccine design under the premise that organisms evolve under diverse environmental conditions that influence their genetic variability [88,89,90]. This ecological and genetic evolution should be considered a central axis in this research field, integrating approaches that combine ecology, genetics, and geography, alongside innovative hypotheses. A similar proposal has recently been put forward, reinforcing the need for integrative approaches to address current challenges and develop more effective vaccines tailored to the environmental and evolutionary contexts of these pathogens [91].

This approach is particularly relevant because, as evidenced in this study, the identification of subunit-based vaccine candidates using in silico tools has become widespread in recent years. Therefore, incorporating these integrative perspectives into current and future research could improve the identification of promising candidates, which could subsequently be evaluated under controlled experimental conditions and in field settings worldwide.

These proposals are based on the fact that *Babesia* species are parasites with a broad global distribution, reflecting their ability to undergo ecological adaptation alongside their vectors. This plasticity represents an evolutionary advantage that promotes the generation of genetic diversity [92], as well as the emergence of genotypes associated with geographical distribution patterns, as recently reported for this genus [93]. Furthermore, considering cattle populations in different ecological contexts allows for the evaluation of vaccine stability under diverse climatic conditions, which is a key aspect in addressing the thermolability of vaccine formulations, as demonstrated in recent studies [94].

Meta-analyses have acquired a central role in the evaluation of biomedical interventions, including vaccines used in both human and veterinary medicine, by providing integrated quantitative estimates with greater statistical power [95,96,97,98]. In the context of vaccines against bovine babesiosis, this represents the first evaluation of its kind, allowing for the synthesis of evidence generated over several decades under heterogeneous technological platforms.

Despite being a historically well-studied field, a considerable proportion of the identified studies did not meet sufficient methodological or data quality standards for inclusion in the quantitative analysis. The heterogeneity in reporting limited the full integration of the available evidence. This finding highlights the need to standardize methodological criteria and reporting formats in future vaccine trials. Our results are based on the available information and, therefore, the inferences should be interpreted within this context; however, the efficacy and protection patterns observed are consistent and robust.

Although a wide diversity of candidate antigens has been described in *Babesia* species affecting cattle, only a fraction has advanced to formal vaccine evaluation stages. Nevertheless, the results of the present meta-analysis suggest a potential positive relevance, supporting the need to promote their development towards challenge trial stages. In parallel, live-attenuated vaccines continue to show the highest levels of protective efficacy. Recent advances in in vitro culture systems, both with bovine serum and in serum-free conditions [99,100], represent promising technological alternatives that could improve the standardization, safety, and scalability of these vaccines. Our results provide quantitative support for their efficacy; however, further trials are warranted based on the future directions outlined above.

An additional aspect that deserves attention is the representativeness of the bovine breeds included in experimental trials. Most studies have been conducted in European breeds, whereas modern livestock systems increasingly rely on synthetic or crossbred animals adapted to tropical and subtropical conditions. The inclusion of these populations in future studies would allow for a generation of evidence that is more applicable to current production systems.

The absence of detectable heterogeneity in vaccine efficacy for mortality outcomes (Figure 8) suggests that, despite substantial variation in experimental designs and vaccine formulations, most studies consistently report protective effects. This pattern indicates robust vaccine performance across different settings. Nevertheless, the limited reporting of failed or non-protective outcomes may introduce positive reporting bias that should not be overlooked. This finding is consistent with previous meta-analyses in livestock vaccine research [97], although it contrasts with reports showing greater variability in vaccine performance [98].

In contrast, heterogeneity was observed for physiological outcomes such as body temperature and hematocrit (Supplementary Material S7; Figure S10), highlighting context-dependent variability in vaccine responses. Such heterogeneity is likely driven by multiple interacting factors, including *Babesia* strain diversity, host genetic variation, environmental conditions, differences in virulence, antigen dose and formulation, route of administration, as well as variability in immune response measurement and study design. Collectively, these factors underscore the complexity of comparing vaccine performance across heterogeneous experimental and field conditions.

Regarding publication bias, the robustness of both physiological (body temperature and packed cell volume) and mortality outcomes is supported by the absence of significant funnel plot asymmetry detected by Egger’s regression test. However, Begg’s rank correlation test suggested significant asymmetry for the temperature outcome, indicating potential small-study effects. This pattern may reflect heterogeneity in experimental design, *Babesia* strain diversity, and challenge protocols rather than selective publication alone. In contrast, for mortality outcomes, statistical testing ruled out significant publication bias, and the trim-and-fill procedure did not impute missing studies, suggesting that the available literature provides a reliable representation of vaccine efficacy. The lack of heterogeneity among mortality trials (I^2^ = 0%) further supports the conclusion that vaccination consistently reduces mortality across diverse experimental settings.

Finally, we propose the development of standardized guidelines for vaccine trials against bovine babesiosis, including harmonized criteria for experimental design, outcome definition, and immunological metrics. The systematic incorporation of comparable immunological variables, such as class-specific antibody titers and CD4+ T cell-mediated responses, would facilitate more robust integrated analyses and improve the understanding of the correlates of protection.

## 5. Future Perspectives and a Roadmap for Next-Generation Vaccines

While reverse vaccinology has rightly become the dominant paradigm, the next decade of research must focus on translating candidate antigens into effective, deployable, and broadly protective vaccines. Our analysis points to several critical frontiers that will define the future of the field:

Overcoming Antigenic Diversity and Enhancing Immunogenicity: The challenge of genetic diversity necessitates moving beyond single-antigen formulations. The future lies in multi-epitope vaccines, rationally designed to include conserved, T-cell and B-cell epitopes from multiple antigens. This approach can provide broad protection against diverse parasite strains. To deliver these constructs effectively, emerging platforms like nanoparticle-based delivery systems (e.g., virus-like particles and lipid nanoparticles) show immense promise. These systems not only enhance antigen presentation and stability but can also be co-delivered with novel adjuvants to skew the immune response towards a more potent and durable Th1-type immunity, which is critical for intracellular parasites like *Babesia*.

The Power of Predictive Integration: AI and Multi-Omics: The sheer volume of genomic, proteomic, and epidemiological data now available demands a shift to computational-powered discovery. The integration of artificial intelligence (AI) and machine learning with multi-omics data will be transformative. AI algorithms can mine complex datasets to predict novel, conserved epitopes, model host–parasite–vector interactions, and even identify correlates of protection, dramatically accelerating the antigen screening pipeline. This “big data” approach will enable the design of vaccines that are not only effective in the lab but also resilient in the face of pathogen evolution.

A Proactive Response to a Changing World: Climate and Vector Expansion: Future vaccine development cannot operate in an ecological vacuum. Climate change is rapidly altering the distribution of tick vectors, exposing naive cattle populations in new regions and potentially altering transmission dynamics in endemic areas [93,94]. A 5-to-10-year research roadmap must therefore prioritize:

- Climate-Resilient Formulations: Developing thermostable vaccine formulations that do not rely on a cold chain is essential for deployment in remote and expanding endemic areas.
- Geographically Tailored Vaccines: Establishing regional surveillance networks to monitor the emergence of new *Babesia* strains and genotypes, informing the continuous update of vaccine candidates to ensure regional relevance.
- Pre-emptive Vaccination Strategies: Using ecological niche modeling to predict future outbreak zones and guide pre-emptive vaccination campaigns, rather than reactive control.

By embracing these integrated platforms and a proactive, ecologically informed strategy, the next generation of bovine babesiosis vaccines can transition from being merely conceptual to being broadly effective, readily deployable, and resilient tools for global cattle health.

## 6. Conclusions and Future Directions

This systematic and scientometric review provides the first comprehensive, global synthesis of research in six decades on vaccine development against bovine babesiosis. Our findings highlight the predominance of *B. bovis* as the focus of vaccine research, despite the comparable or broader distribution of *B. bigemina* and the epidemiological importance of *B. divergens* and *B. major* in certain regions. Over time, methodological trends have shifted from field and controlled challenge trials to approaches centered on in silico antigen identification, immunogenicity evaluation, and in vitro assays, in line with the principles of reverse vaccinology.

The field now stands on the brink of a new era, driven by technological convergence. To accelerate progress, future research must:

- Leverage Advanced Platforms: Prioritize the development of multi-epitope vaccines using delivery systems that overcome antigenic diversity and enhance immunogenicity.
- Embrace Data-Driven Discovery: Integrate AI and machine learning with multi-omics data to refine epitope prediction, identify correlates of protection, and guide rational vaccine and adjuvant design.
- Adopt an Eco-Epidemiological Framework: Address the challenge of climate change by developing thermostable, geographically informed vaccines and using ecological modeling to guide pre-emptive vaccination strategies in areas of vector expansion.

Ultimately, strengthening international collaborations—particularly by including underrepresented, high-impact regions in Africa—and fostering transdisciplinary partnerships between molecular biologists, bioinformaticians, ecologists, and veterinarians will be paramount. By addressing these priorities, the next generation of bovine babesiosis vaccines can offer greater effectiveness, regional adaptability, and a substantial contribution to global cattle health and productivity in a rapidly changing world.

## Data Availability

The original contributions presented in this study are included in the article/Supplementary Material. Further inquiries can be directed to the corresponding authors.

## Supplementary Materials

The following supporting information can be downloaded at https://www.mdpi.com/article/10.3390/pathogens15050500/s1. Supplementary Material S1: Table .csv with all studies included in this systematic review; Supplementary Material S2: Bibliographic references of this systematic review in PubMed and Web of Science formats; Supplementary Material S3: *Babesia* species considered in vaccine development over time, based on the publications selected in this study; Supplementary Material S4: A graphic showing the number of publications per country on the bovine babesiosis vaccine; Supplementary Material S5: Additional information on bibliometric and scientometric analysis; Figure S1. Annual scientific production; Figure S2. Most relevant sources; Figure S3. Core sources; Figure S4. Most locally cited sources; Figure S5. Most relevant authors; Figure S6. Author production over time; Figure S7. Corresponding authors’ countries; Figure S8. Country scientific production; Figure S9. Country collaboration map; Figure S10. Country production over time; Figure S11. Affiliations and production over time; Figure S12. Most relevant affiliations; Figure S13. Most relevant words in the keywords; Figure S14. Most relevant words in the titles; Figure S15. Co-occurrence network; Figure S16. Co-occurrence network from authors’ keywords; Figure S17. Co-occurrence network from the titles; Figure S18. Co-occurrence network from the titles; Figure S19. Co-occurrence network from the titles; Figure S20. Co-occurrence network from the abstract; Figure S21. Thematic map for the year 2025; Figure S22. Thematic evolution on bovine babesiosis vaccine research; Supplementary Material S6: CSV file containing all studies included in the meta-analysis and the data used; Supplementary Material S7: Figure S1. Forest plot of the sensitivity analysis (leave-one-out); Figure S2. Forest plot showing the comparison of mortality between vaccinated and control groups by vaccine type; Figure S3. Temperature-controlled experiment; Figure S4. Hematocrit-controlled experiment; Figure S5. Temperature field experiment; Figure S6. Temperature subunit vaccine-controlled experiment; Figure S7. Hematocrit subunit vaccine-controlled experiment; Figure S8. Temperature global experiments; Figure S9. Hematocrit global experiments; and Figure S10. Contour-enhanced funnel plots are shown for (A) temperature and (B) hematocrit outcomes (SMD).

## Appendix A

**Section and Topic**	**Item #**	**Checklist Item**	**Page** **#**
TITLE			
Title	1	Identify the report as a systematic review.	1
ABSTRACT			
Abstract	2	The abstract presents the study as a systematic review, including the objective, search sources, methods for synthesis, and the interpretation of the results and discussion.	1
INTRODUCTION			
Rationale	3	Describe the rationale for the review in the context of existing knowledge.	2–3
Objectives	4	Provide an explicit statement of the objective(s) or question the review addresses.	3
METHODS			
Eligibility criteria	5	Specify the inclusion and exclusion criteria for the review and how studies were grouped for the syntheses.	3
Information sources	6	Specify all databases, registers, websites, organizations, reference lists, and other sources searched or consulted to identify studies. Specify the date when each source was last searched or consulted.	3
Search strategy	7	Present the full search strategies for all databases, registers, and websites, including any filters and limits used.	3
Selection process	8	Specify the methods used to decide whether a study met the inclusion criteria of the review, including how many reviewers screened each record and each report retrieved, whether they worked independently, and, if applicable, details of the automation tools used in the process.	3
Data collection process	9	Specify the methods used to collect data from reports, including how many reviewers collected data from each report, whether they worked independently, any processes for obtaining or confirming data from study investigators, and, if applicable, details of the automation tools used in the process.	3–4
Data items	10a	List and define all outcomes for which data were sought. Specify whether all results that were compatible with each outcome domain in each study were sought (e.g., for all measures, time points, analyses), and if not, the methods used to decide which results to collect.	3–4
	10b	List and define all other variables for which data were sought (e.g., participant and intervention characteristics and funding sources). Describe any assumptions made about any missing or unclear information.	3–4
Study risk of bias assessment	11	Specify the methods used to assess the risk of bias in the included studies, including details of the tool(s) used, how many reviewers assessed each study, and whether they worked independently, and, if applicable, details of automation tools used in the process.	3–5
Effect measures	12	Specify for each outcome the effect measure(s) (e.g., risk ratio and mean difference) used in the synthesis or presentation of results.	NA
Synthesis methods	13a	Describe the processes used to decide which studies were eligible for each synthesis (e.g., tabulating the study intervention characteristics and comparing against the planned groups for each synthesis (item #5)).	4
	13b	Describe any methods required to prepare the data for presentation or synthesis, such as handling of missing summary statistics or data conversions.	4
	13c	Describe any methods used to tabulate or visually display the results of individual studies and syntheses.	4
	13d	Describe any methods used to synthesize results and provide a rationale for the choice(s). If meta-analysis was performed, describe the model(s), method(s) to identify the presence and extent of statistical heterogeneity, and software package(s) used.	4
	13e	Describe any methods used to explore possible causes of heterogeneity among study results (e.g., subgroup analysis and meta-regression).	4
	13f	Describe any sensitivity analyses conducted to assess the robustness of the synthesized results.	4
Reporting bias assessment	14	Describe any methods used to assess the risk of bias due to missing results in a synthesis (arising from reporting biases).	4–5
Certainty assessment	15	Describe any methods used to assess certainty (or confidence) in the body of evidence for an outcome.	4
RESULTS			
Study selection	16a	Describe the results of the search and selection process, from the number of records identified in the search to the number of studies included in the review, ideally using a flow diagram.	4–5
	16b	Cite studies that might appear to meet the inclusion criteria, but which were excluded, and explain why they were excluded.	5–10
Study characteristics	17	Cite each included study and present its characteristics.	5–10
Risk of bias in studies	18	Present assessments of the risk of bias for each included study.	5–10
Results of individual studies	19	For all outcomes, present for each study: (a) summary statistics for each group (where appropriate) and (b) an effect estimate with its precision (e.g., confidence/credible interval), ideally using structured tables or plots.	11–15
Results of syntheses	20a	For each synthesis, briefly summarize the characteristics and the risk of bias among contributing studies.	15
	20b	Present the results of all statistical syntheses conducted. If meta-analysis was done, present for each the summary estimate and its precision (e.g., confidence/credible interval) and measures of statistical heterogeneity. If comparing groups, describe the direction of the effect.	11–15
	20c	Present the results of all investigations of possible causes of heterogeneity among study results.	5–10
	20d	Present the results of all sensitivity analyses conducted to assess the robustness of the synthesized results.	5–10
Reporting biases	21	Present the assessments of the risk of bias due to missing results (arising from reporting biases) for each synthesis assessed.	5–10
Certainty of evidence	22	Present the assessments of certainty (or confidence) in the body of evidence for each outcome assessed.	5–10
DISCUSSION			
Discussion	23a	Provide a general interpretation of the results in the context of other evidence.	15–20
	23b	Discuss any limitations of the evidence included in the review.	15–20
	23c	Discuss any limitations of the review processes used.	15–20
	23d	Discuss implications of the results for practice, policy, and future research.	15–20
OTHER INFORMATION			
Registration and protocol	24a	Provide registration information for the review, including register name and registration number, or state that the review was not registered.	NA
	24b	Indicate where the review protocol can be accessed or state that a protocol was not prepared.	NA
	24c	Describe and explain any amendments to the information provided at registration or in the protocol.	NA
Support	25	Describe the sources of financial or non-financial support for the review and the role of the funders or sponsors in the review.	20
Competing interests	26	Declare any competing interests of review authors.	20
Availability of data, code, and other materials	27	Report which of the following are publicly available and where they can be found: template data collection forms; data extracted from included studies; data used for all analyses; analytic code; any other materials used in the review. (See supplementary materials)	20
