# Supplementary material for: Six Decades of Global Research on Bovine Babesiosis Vaccines: A Comprehensive Systematic Review and Meta-Analysis
Source: Pathogens. 2026 May 6;15(5):500. doi: 10.3390/pathogens15050500 (PMC13209936; doi:10.3390/pathogens15050500)
Supplement: Supplementary file 1 [file pathogens-15-00500-s001.zip › Supplementary_Material_S5.pdf]

Supplementary material about bibliometric and scientometric analysis

Title: Six Decades of Global Research on Bovine Babesiosis Vaccines: A Comprehensive Systematic Review and Meta-Analysis

Authors: Uriel Mauricio Valdez-Espinoza, Chyntia Pérez-Almeida, Alma Cárdenas-Flores, Edwin Esaú Hernández-Arvizu and Juan Mosqueda

Date: September 2025

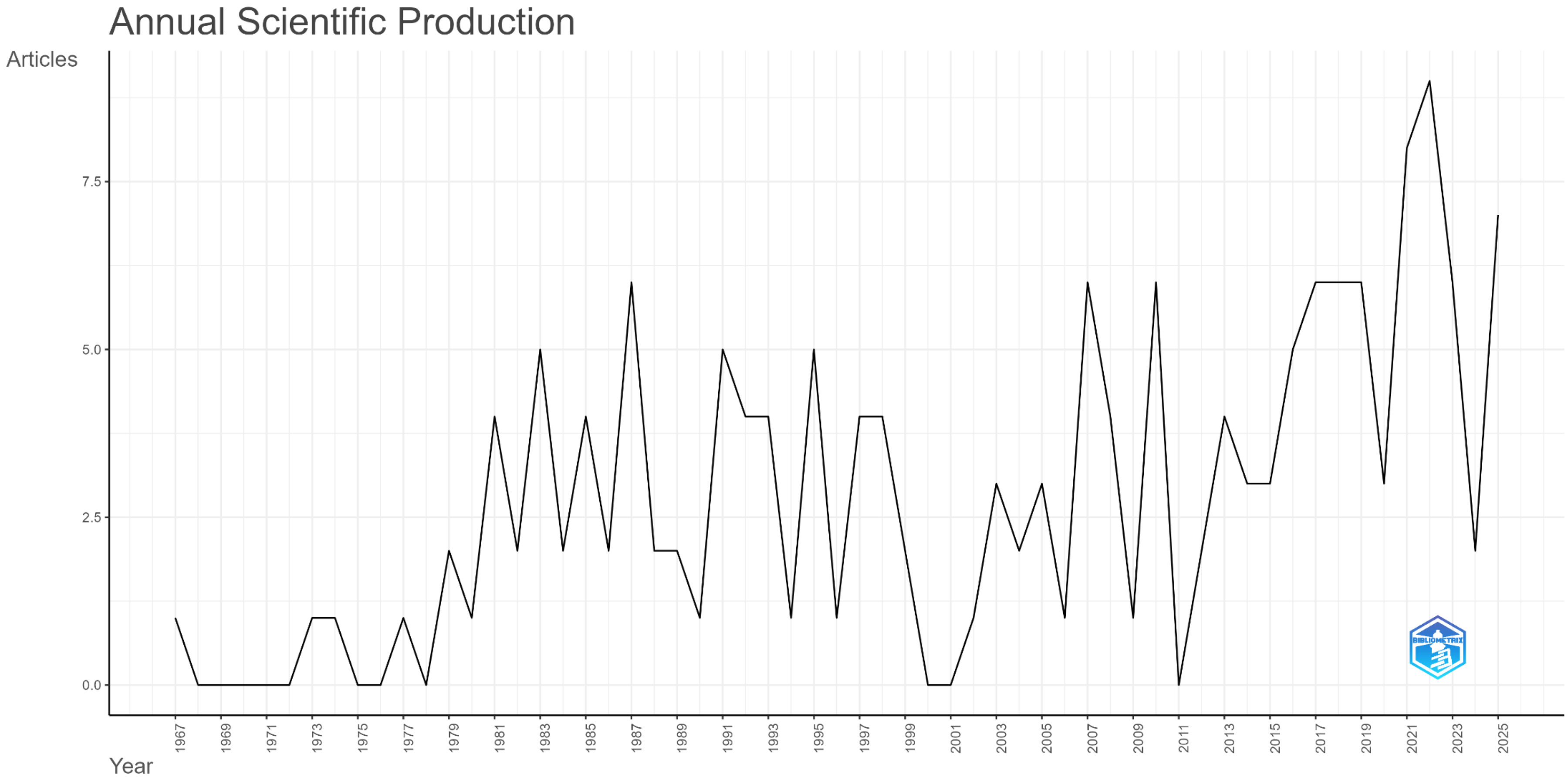

Figure S1. Annual scientific production. Elaboration with bibliographic references format originated in PubMed.

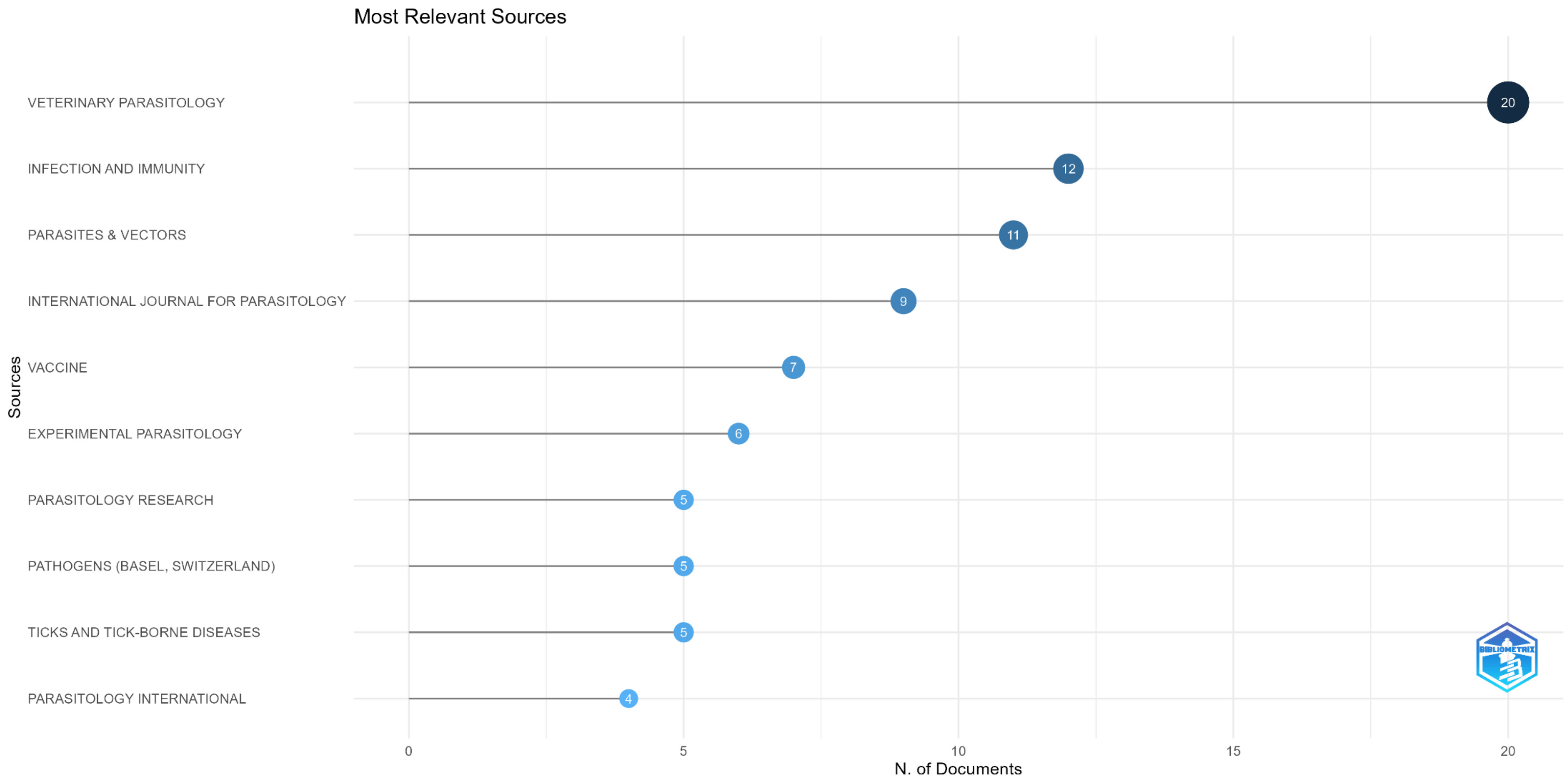

Figure S2. Most relevant sources. Elaboration with bibliographic references format originated in PubMed.

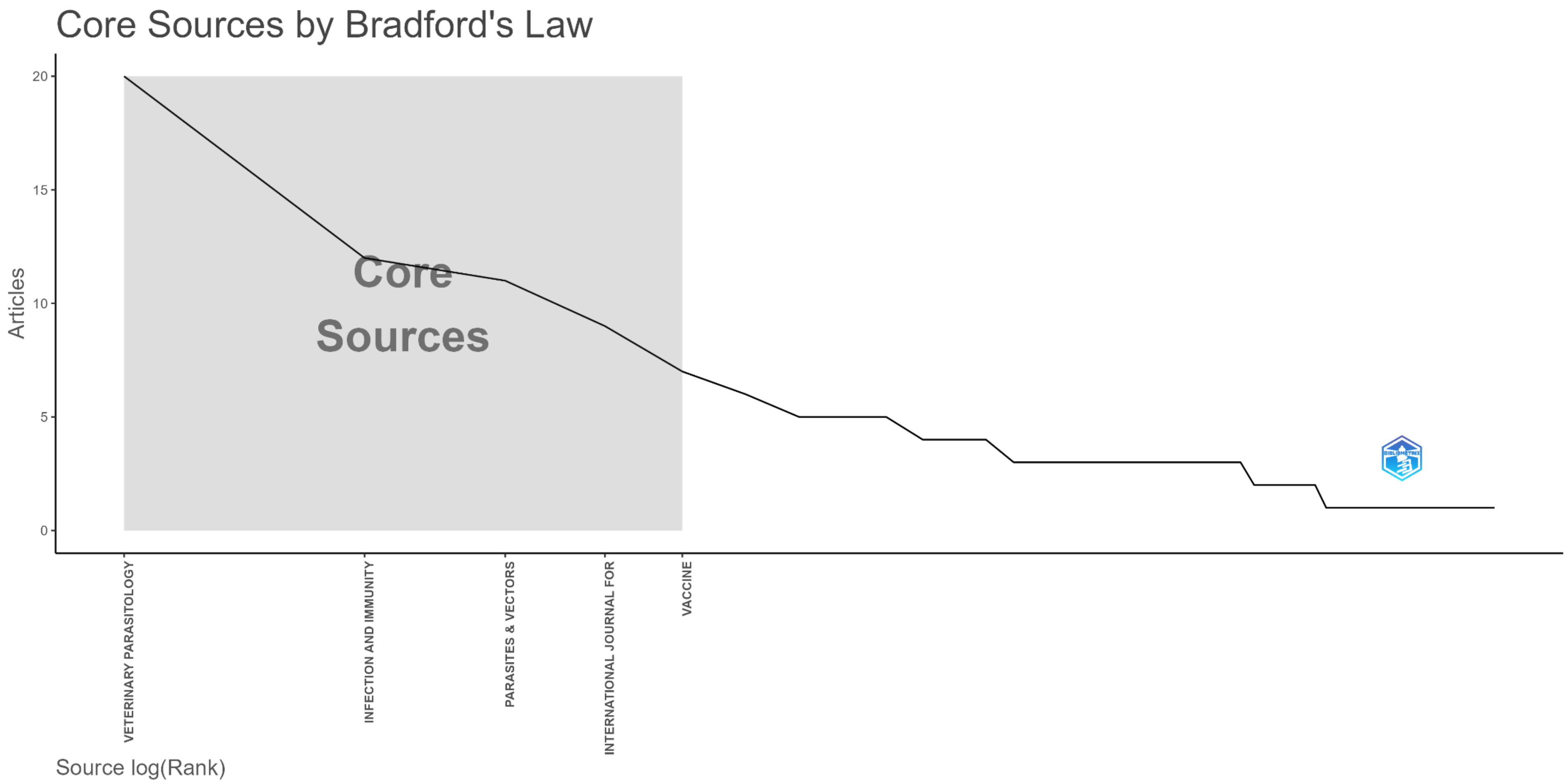

Figure S3. Core sources. Elaboration with bibliographic references format originated in PubMed.

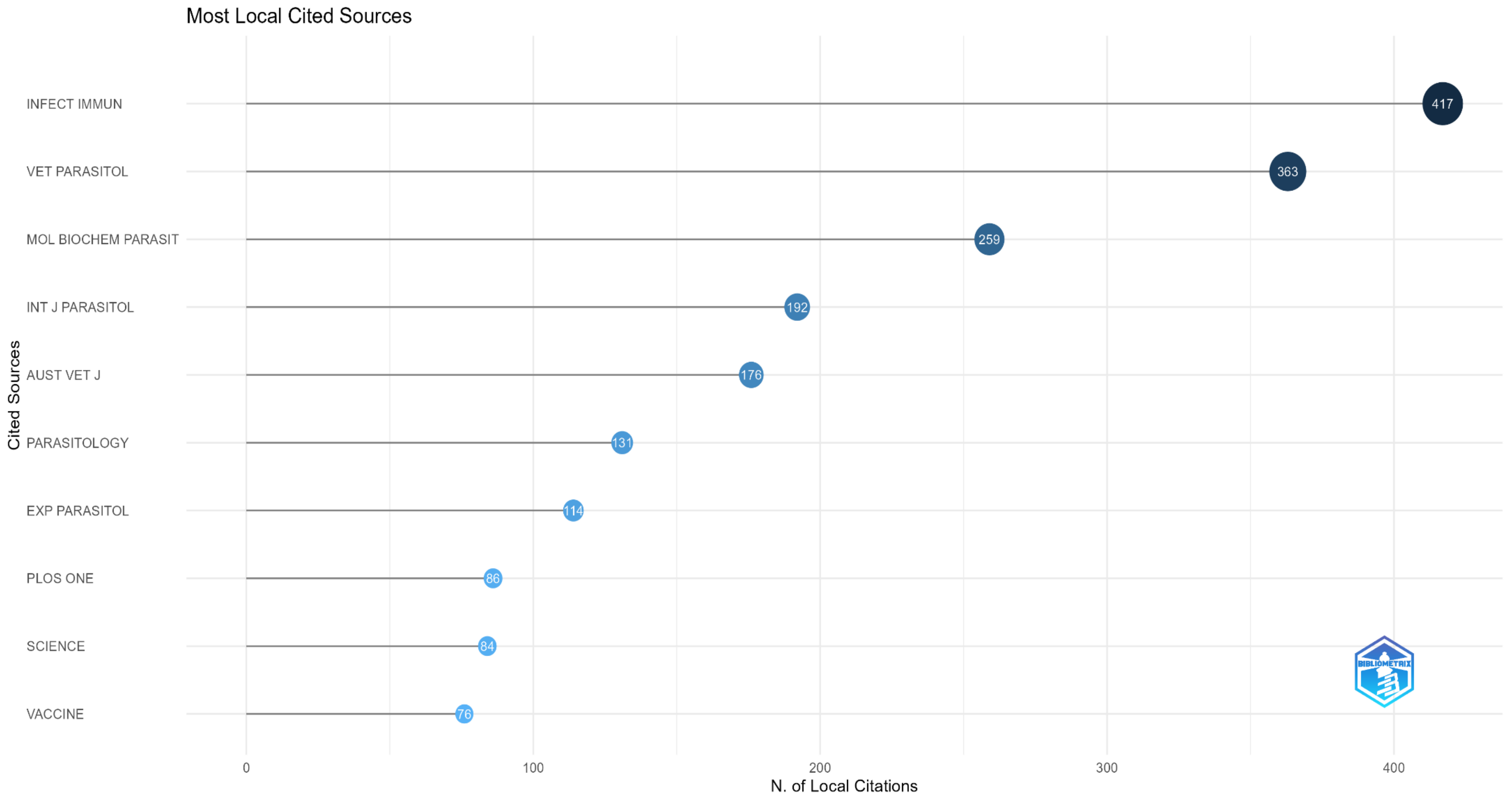

Figure S4. Most local cited sources. Elaboration with bibliographic references format originated in PubMed.

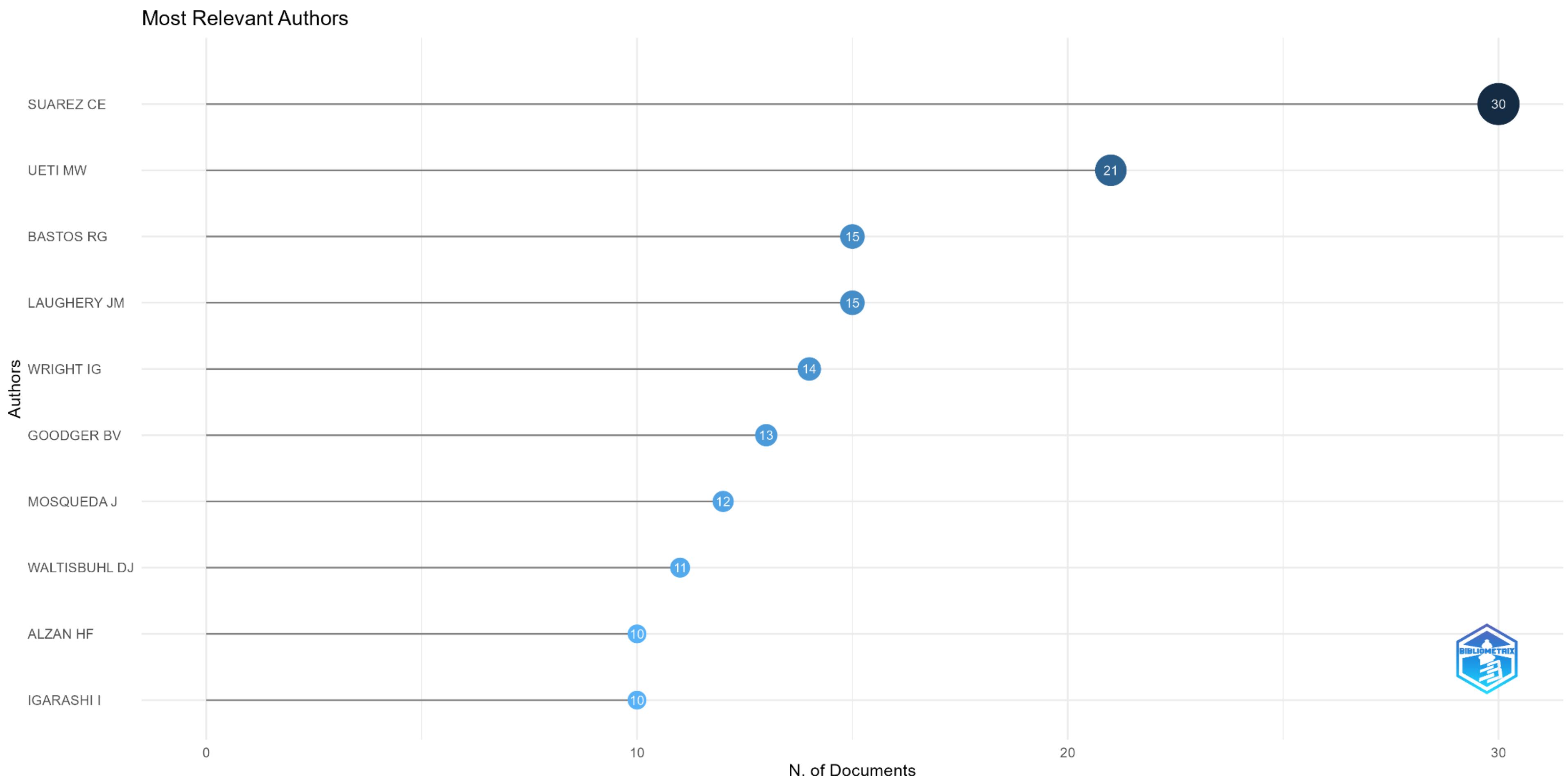

Figure S5. Most relevant authors. Elaboration with bibliographic references format originated in PubMed.

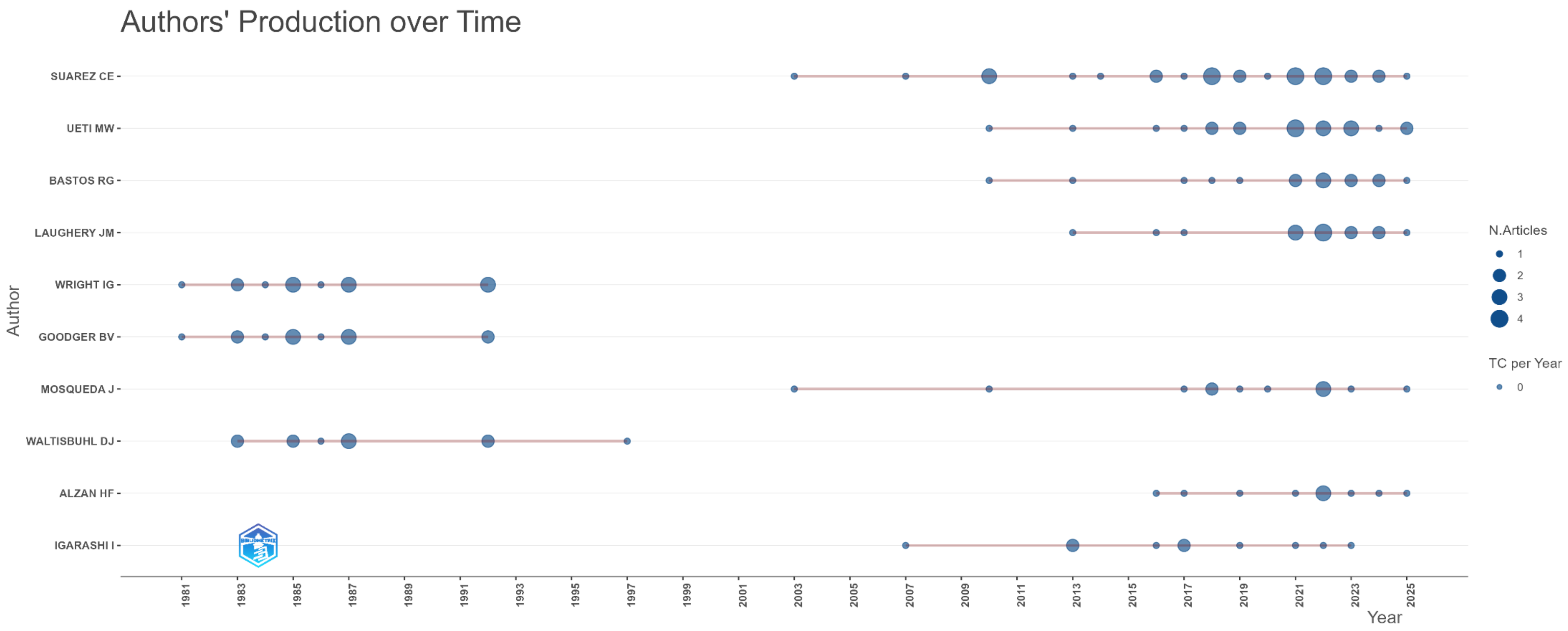

Figure S6. Author Production over time. Elaboration with bibliographic references format originated in PubMed.

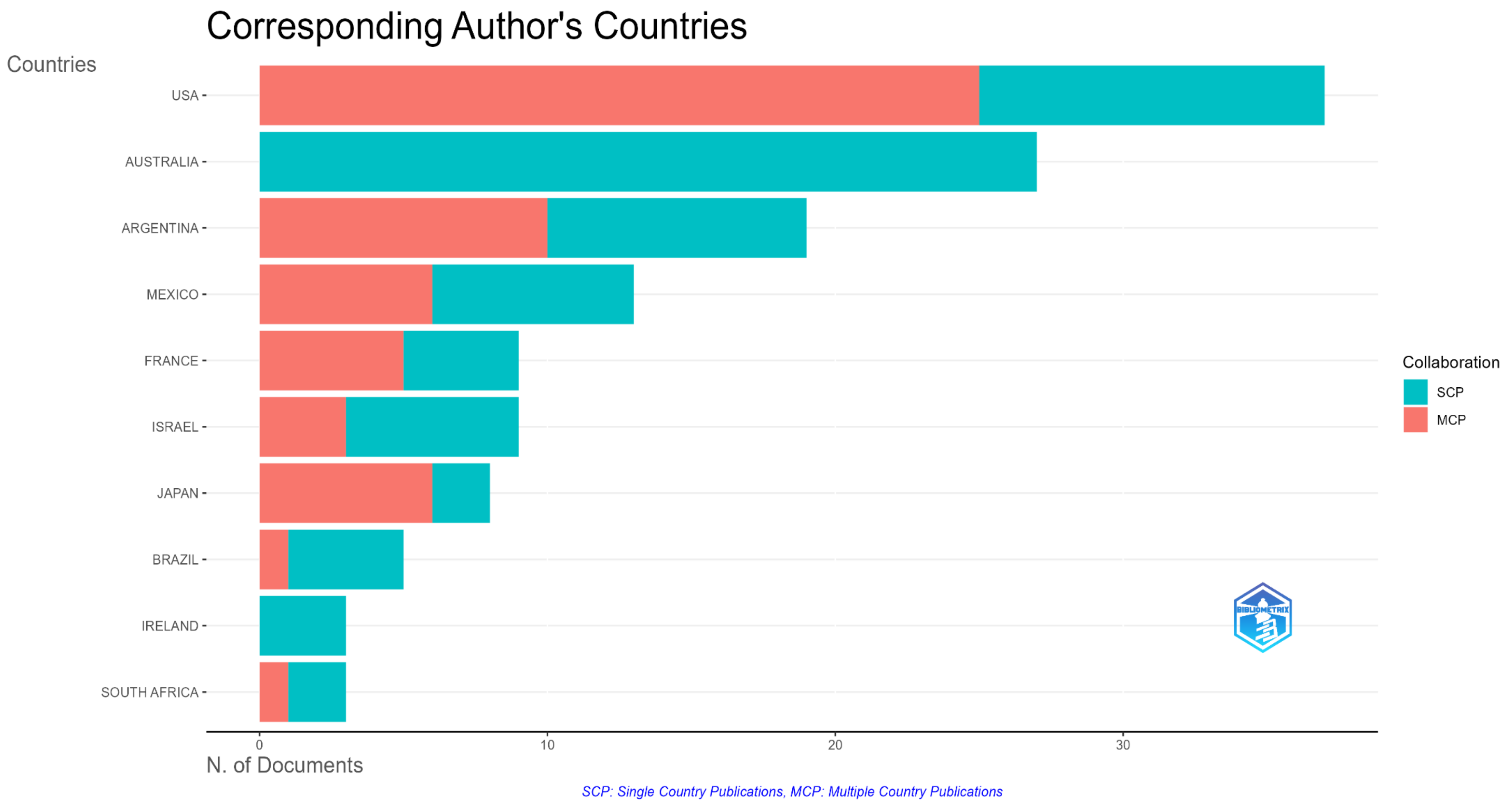

Figure S7. Corresponding author´s countries. Elaboration with. bib originated in Web of Science

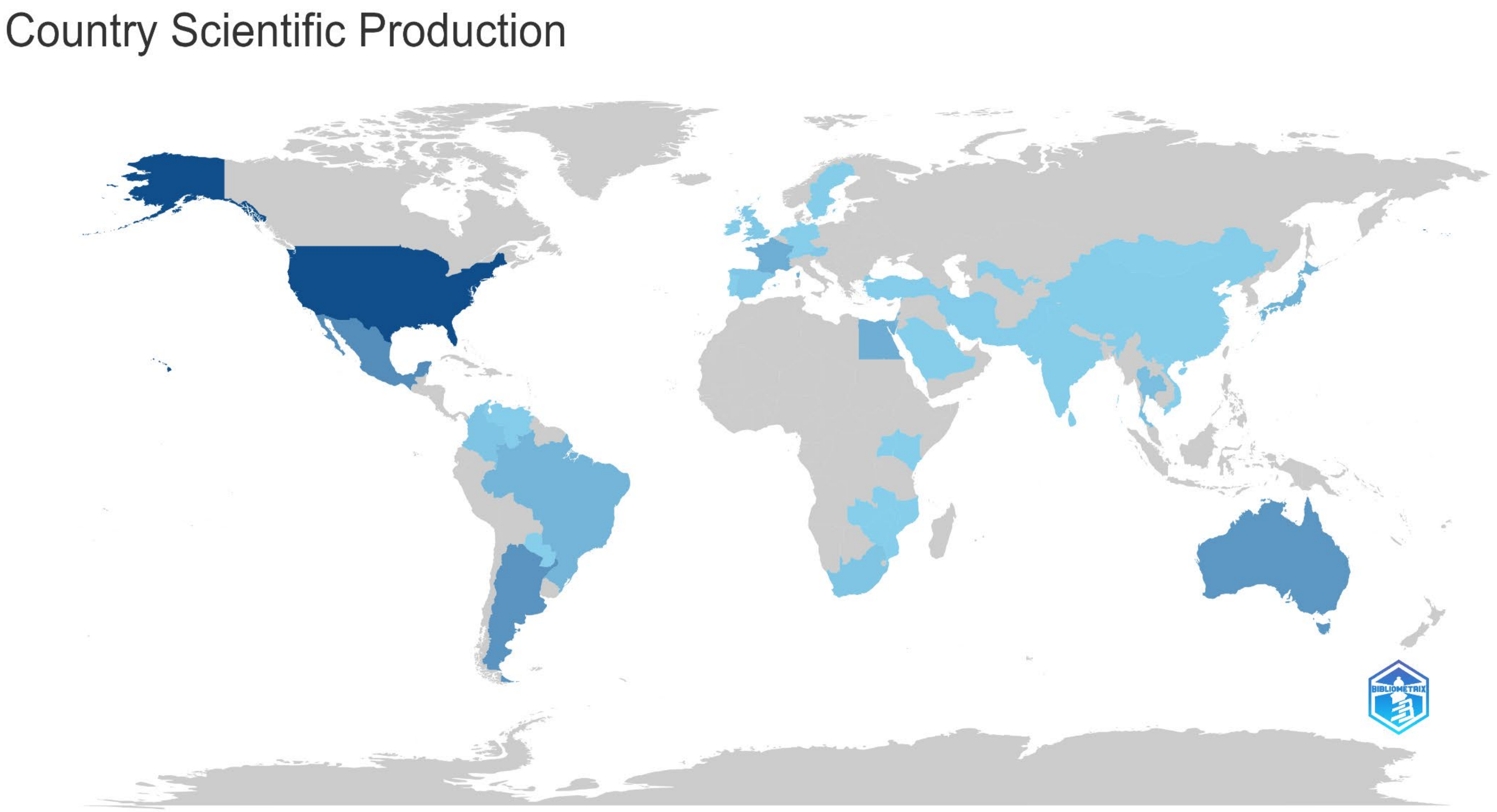

Figure S8. Country scientific production. Elaboration with. bib originated in Web of Science

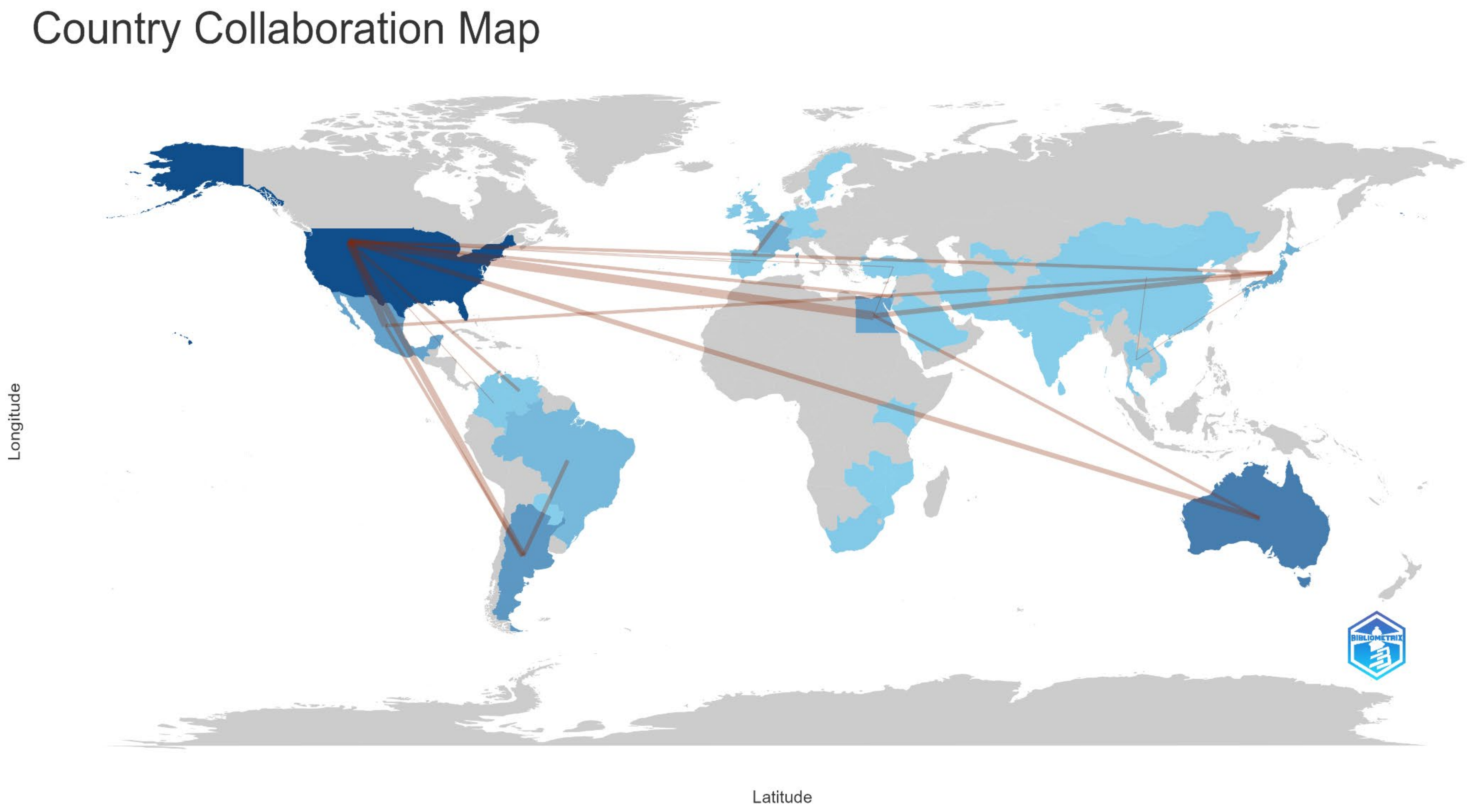

Figure S9. Country collaboration map. Elaboration with. bib originated in Web of Science

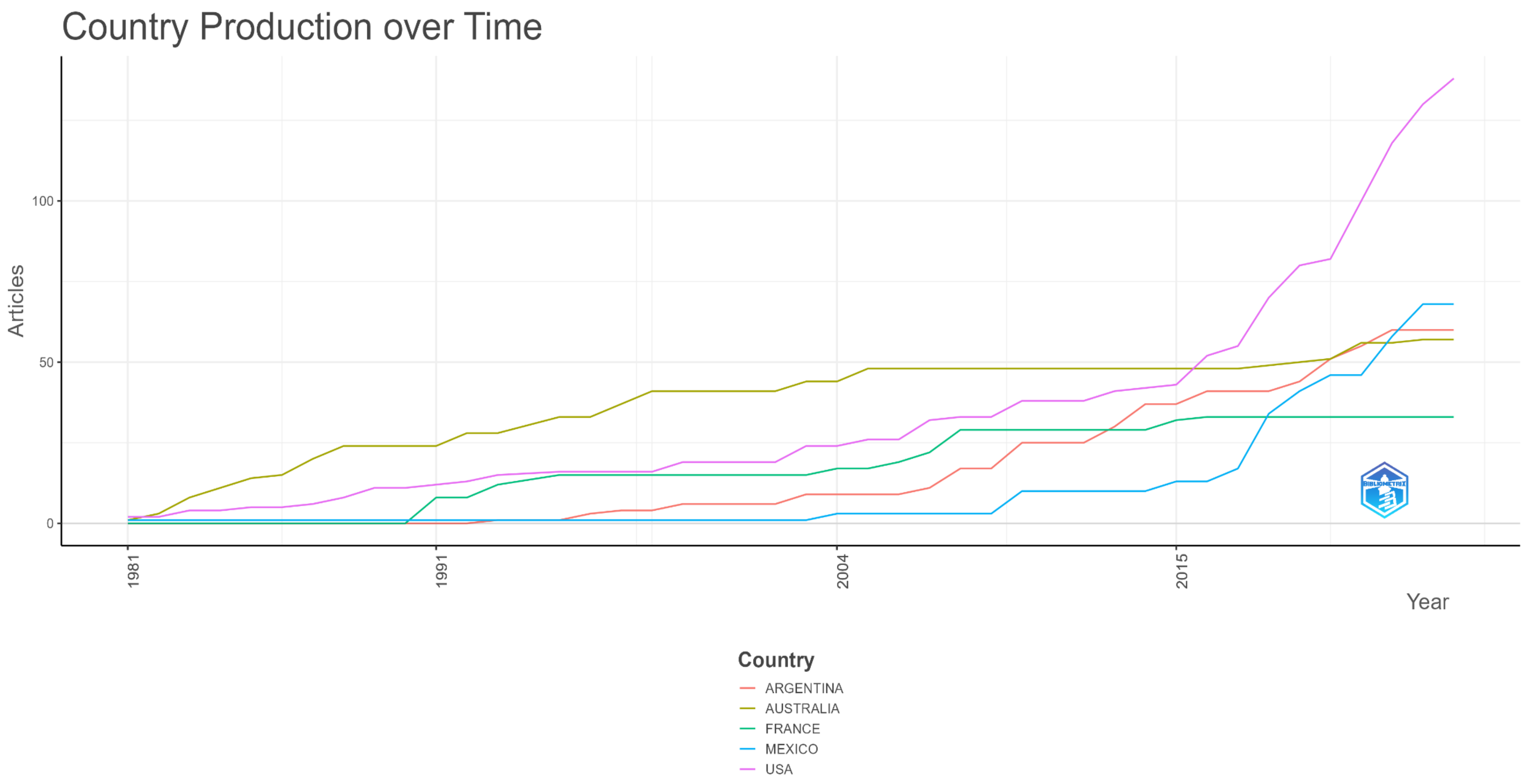

Figure S10. Country production over time. Elaboration with. bib originated in Web of Science

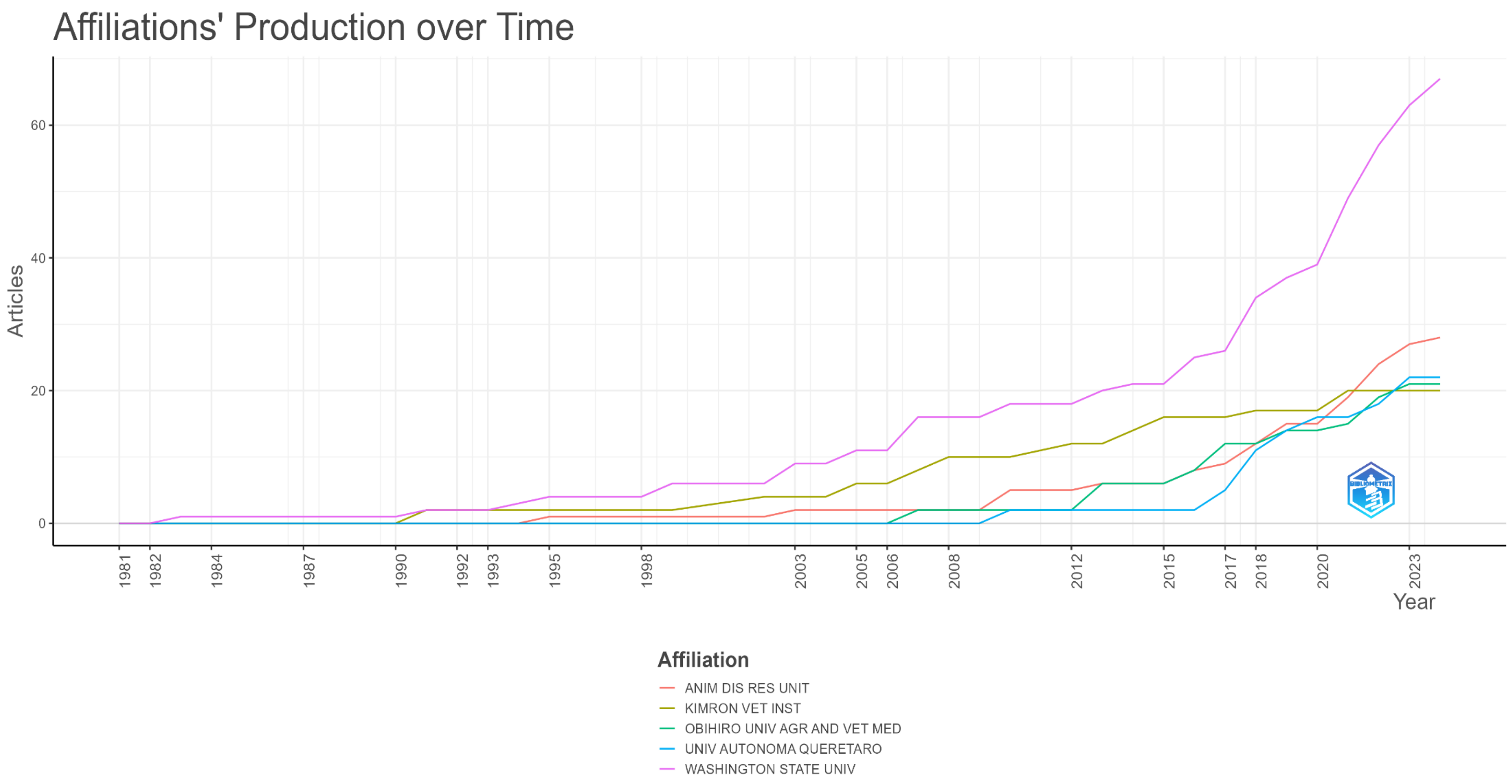

Figure S11. Affiliations´production over time. Elaboration with. bib originated in Web of Science

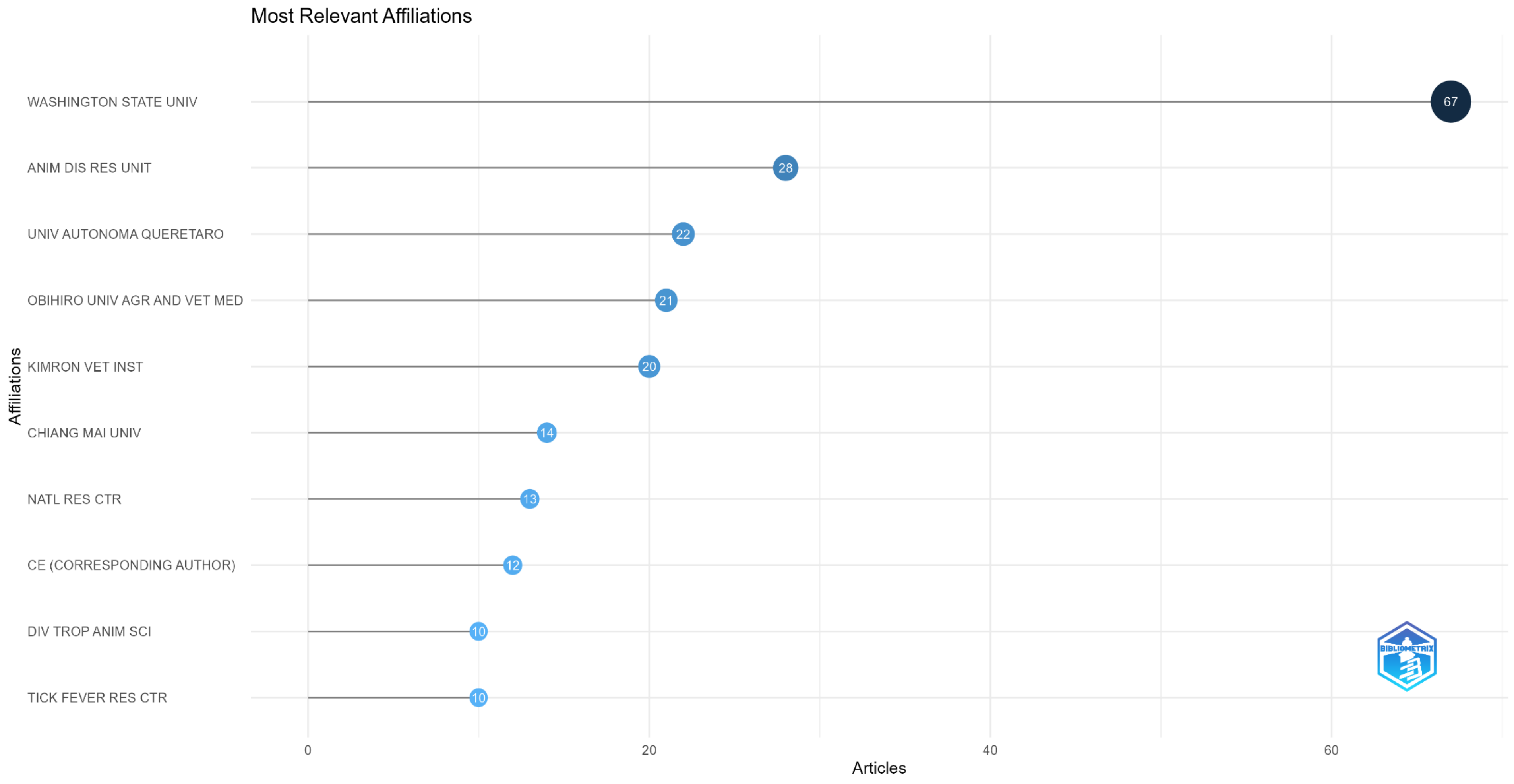

Figure S12. Most relevant affiliations. Elaboration with. bib originated in Web of Science

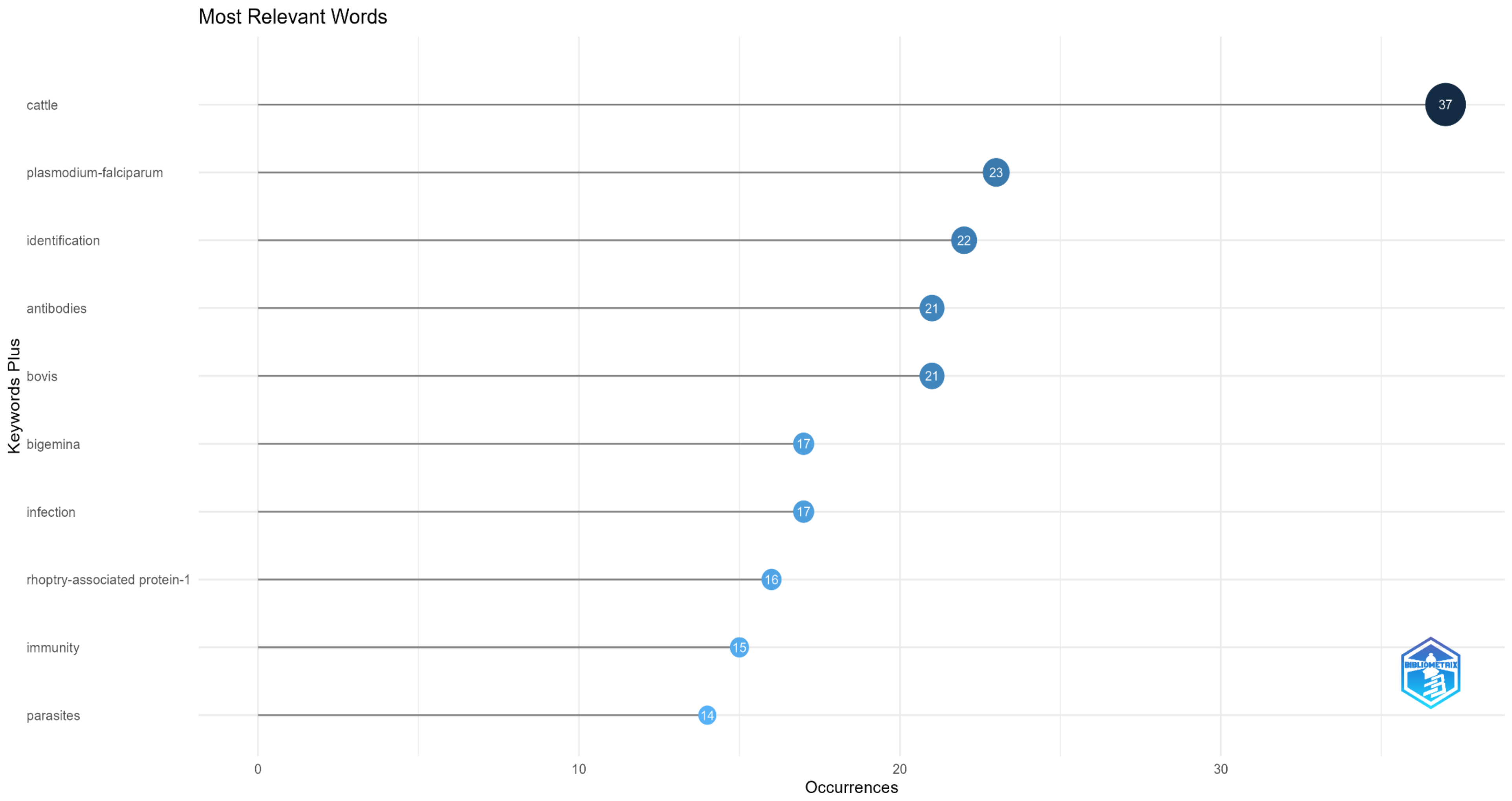

Figure S13. Most relevant words in the keywords plus. Elaboration with. bib originated in Web of Science

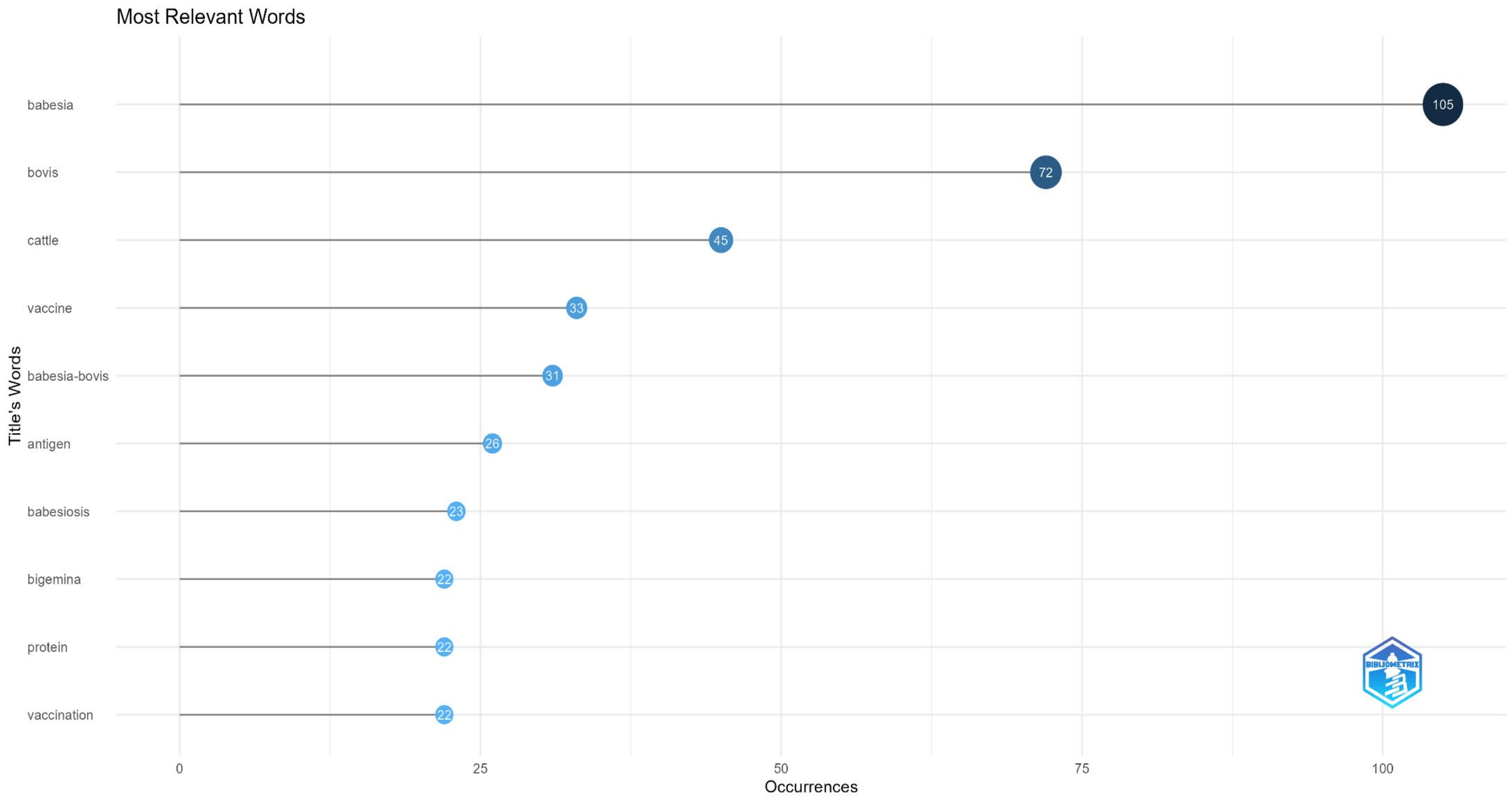

Figure S14. Most relevant words in the titles. Elaboration with. bib originated in Web of Science

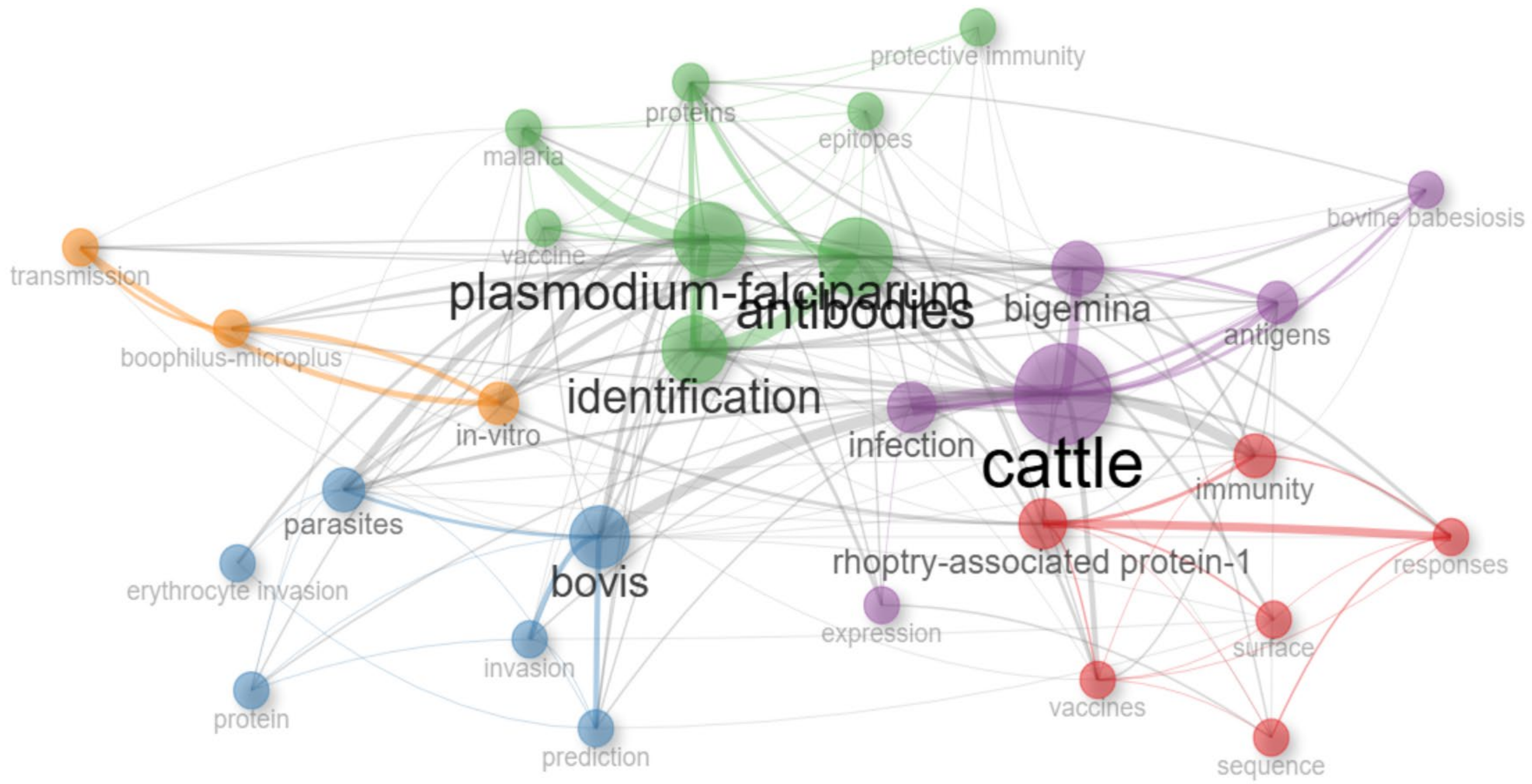

Figure S15. Cooccurrence network. Elaboration with. bib originated in Web of Science

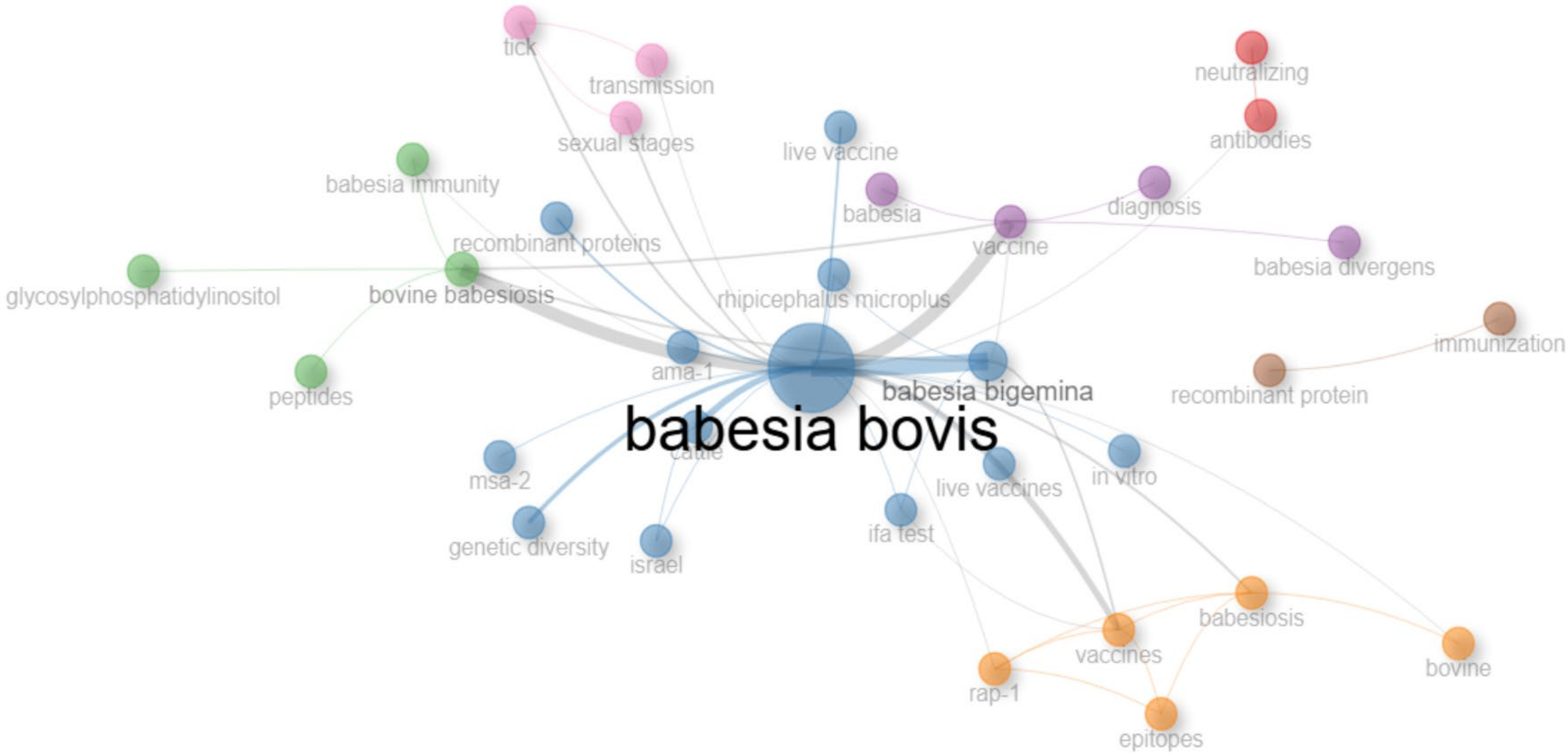

Figure S16. Cooccurrence network from author´s keyword. Elaboration with. bib originated in Web of Science

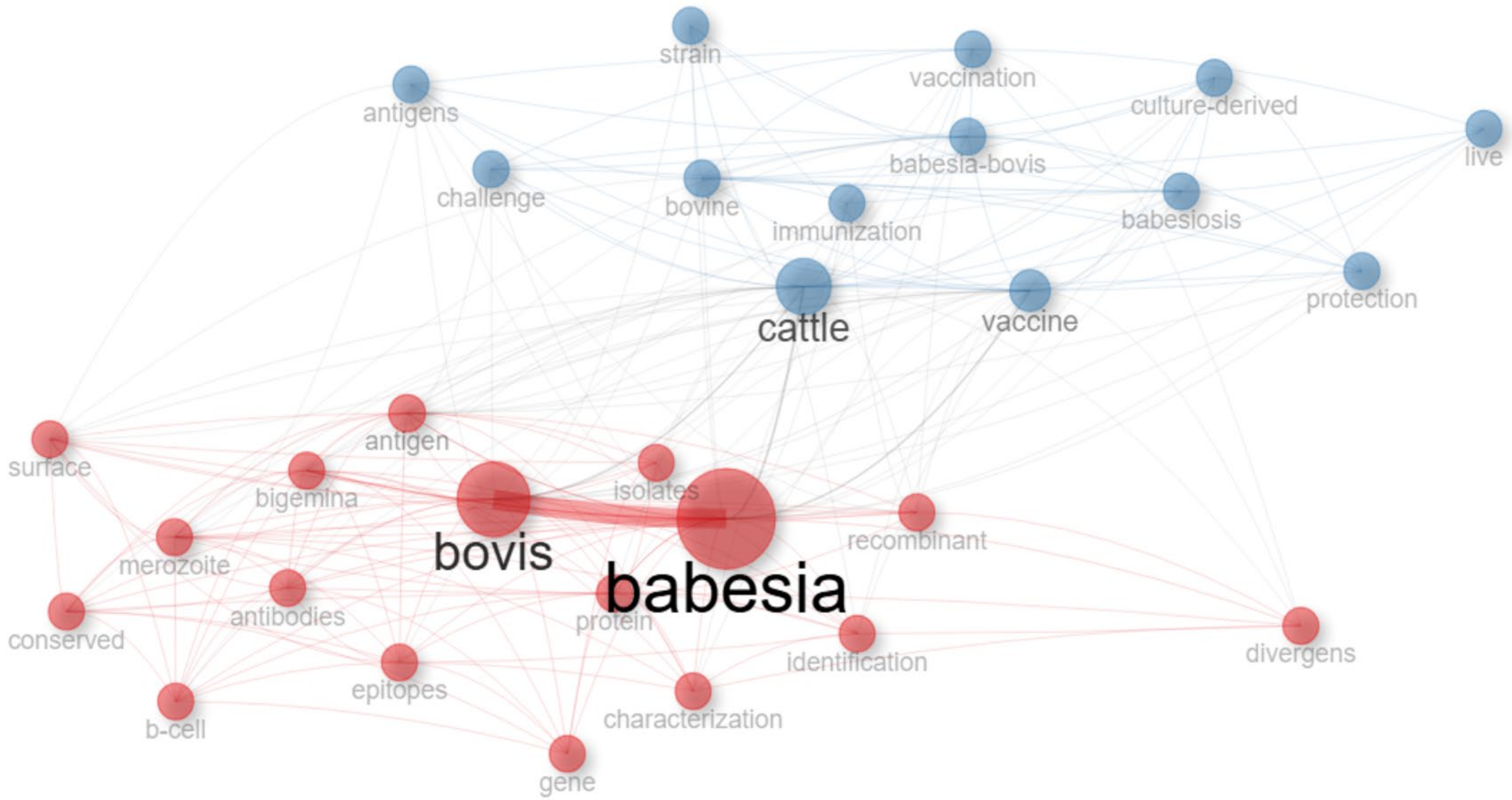

Figure S17. Cooccurrence network from titles. Elaboration with. bib originated in Web of Science

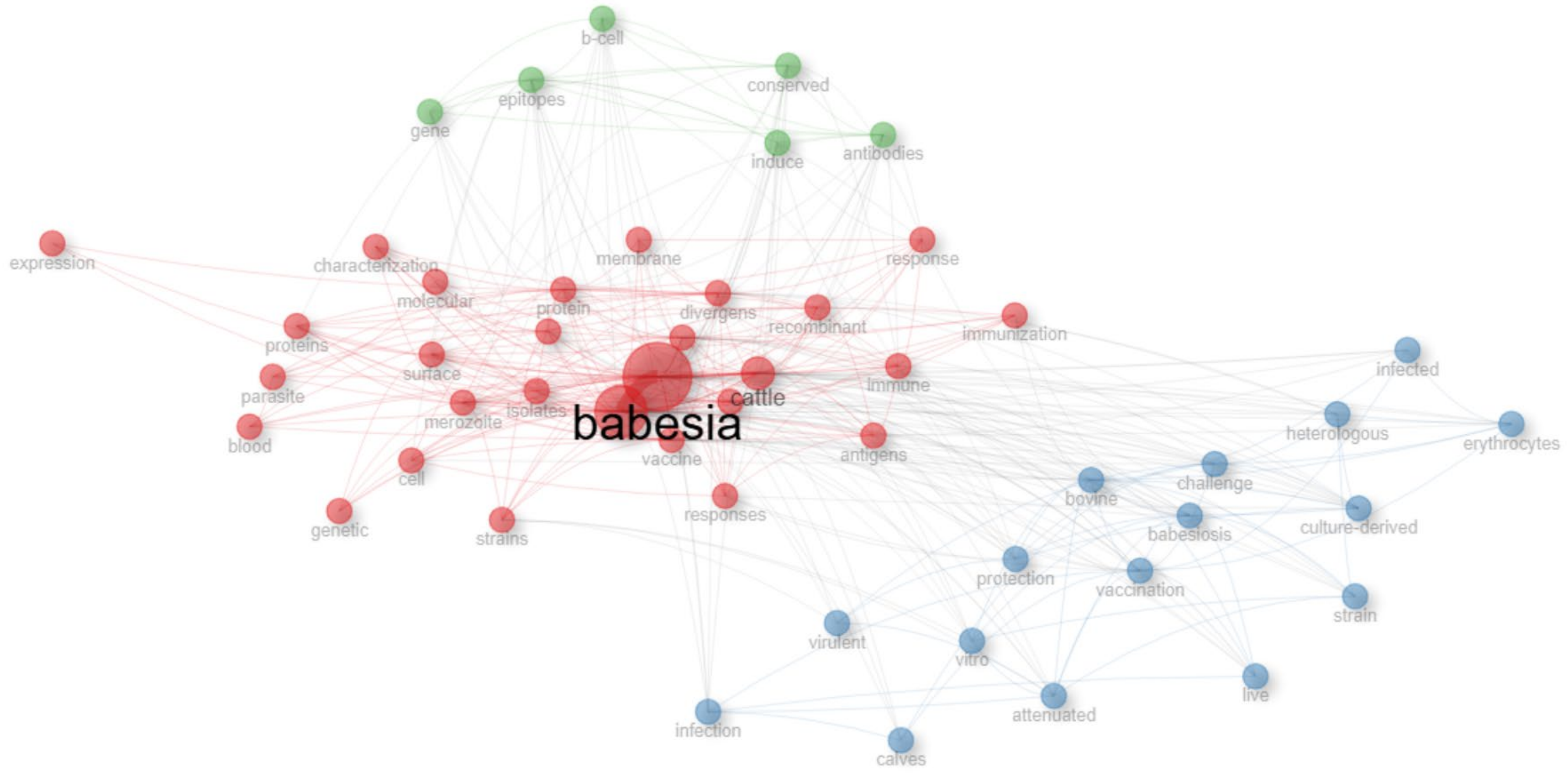

Figure S18. Cooccurrence network from titles. Elaboration with. bib originated in PubMed

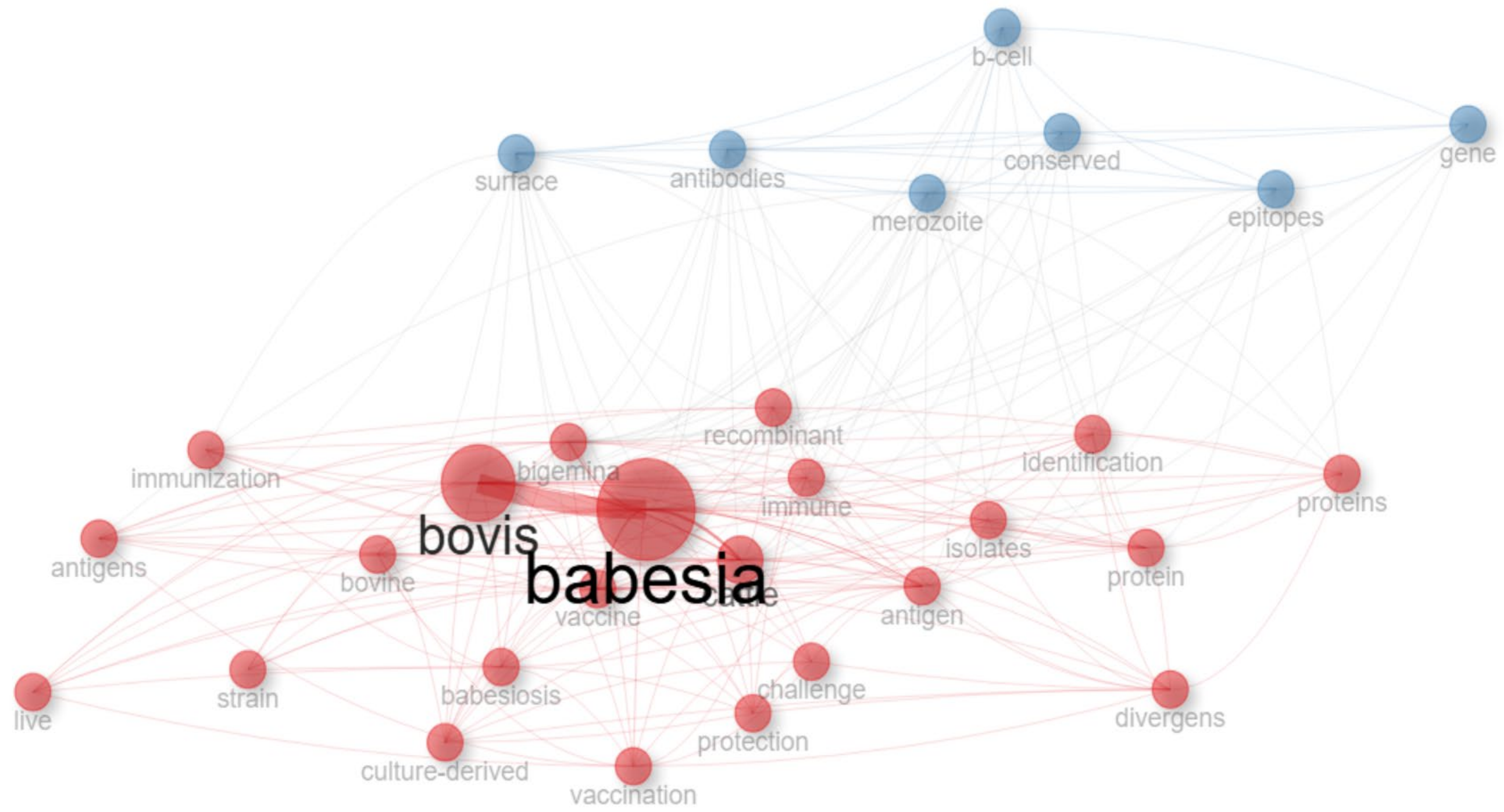

Figure S19. Cooccurrence network from titles. Elaboration with. bib originated in PubMed

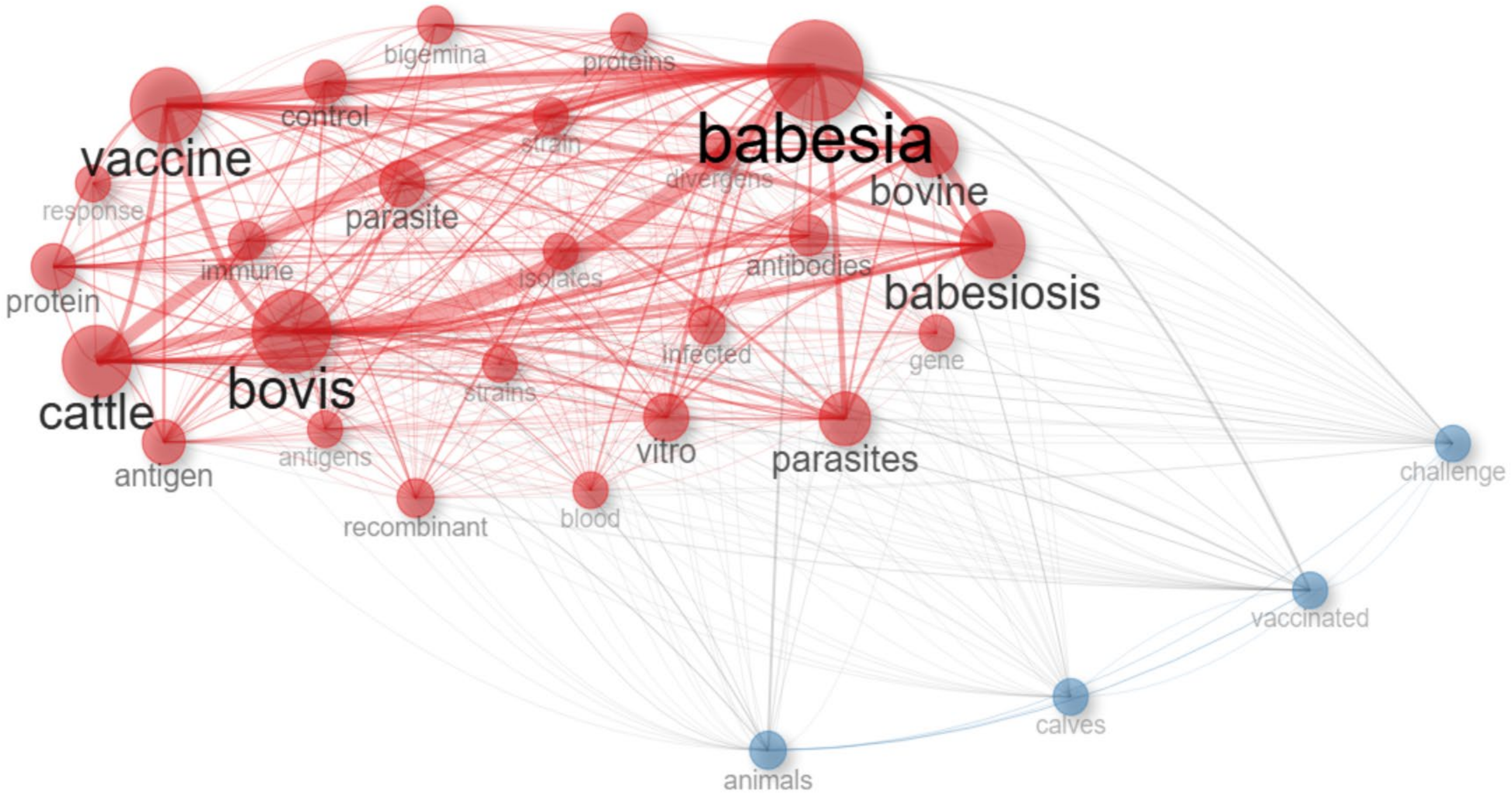

Figure S20. Cooccurrence network from abstract. Elaboration with. bib originated in PubMed

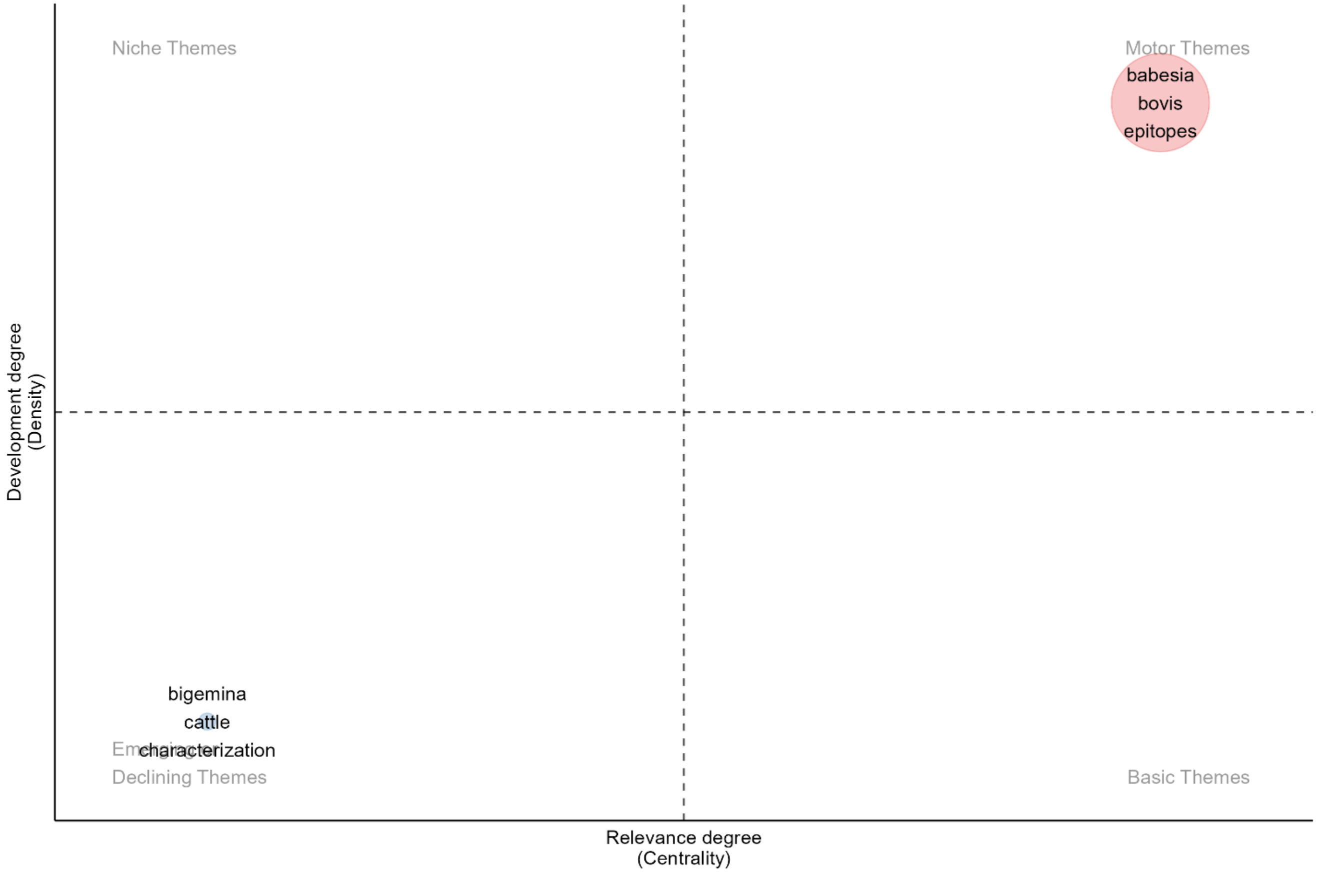

Figure S21. Thematic map for the year 2025. Elaboration with bibliographic references format originated in PubMed.

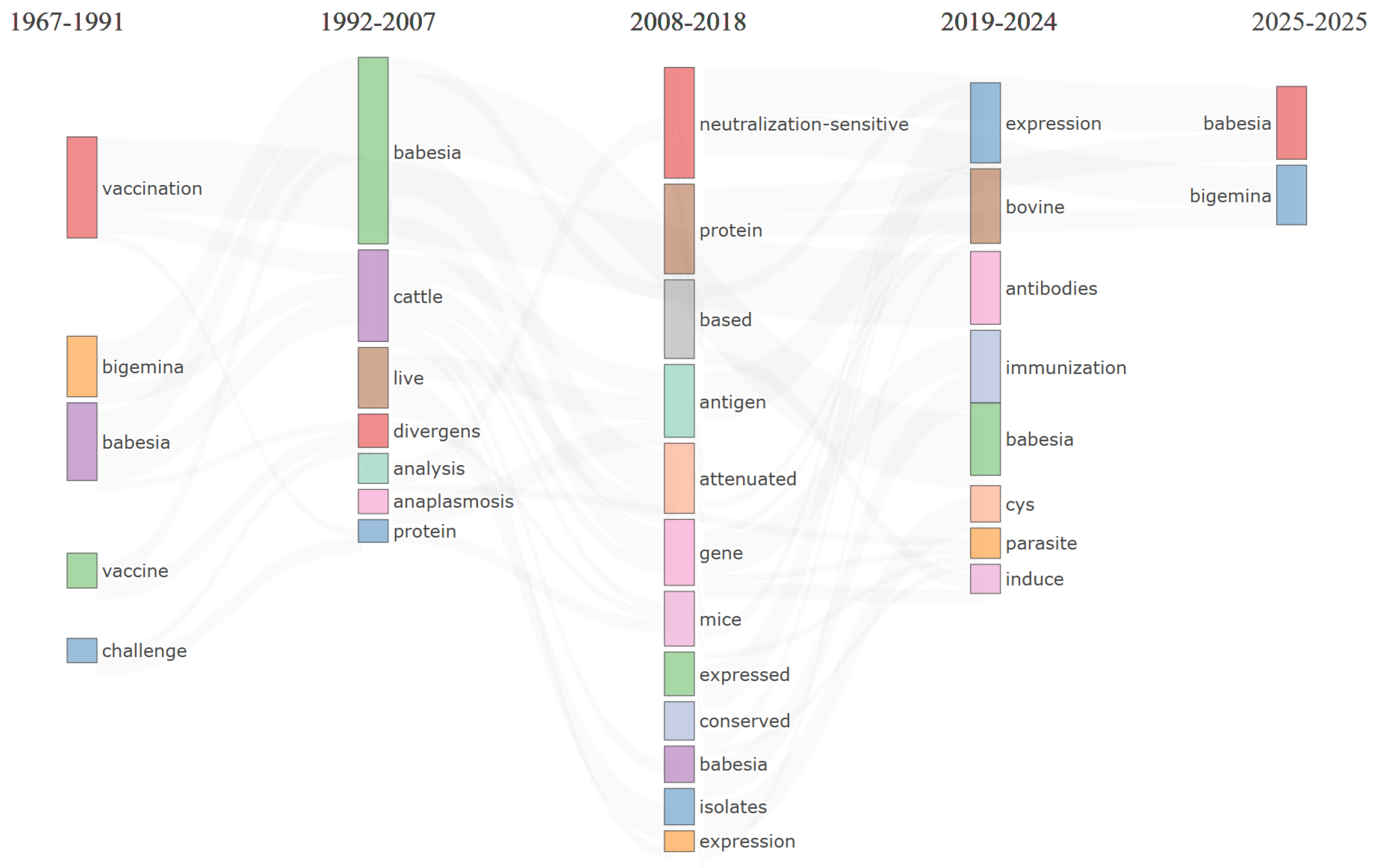

Figure S22. Thematic evolution on bovine babesiosis vaccine research. Elaboration with bibliographic references format originated in PubMed.
