# Supplementary material for: Six Decades of Global Research on Bovine Babesiosis Vaccines: A Comprehensive Systematic Review and Meta-Analysis
Source: Pathogens. 2026 May 6;15(5):500. doi: 10.3390/pathogens15050500 (PMC13209936; doi:10.3390/pathogens15050500)
Supplement: Supplementary file 1 [file pathogens-15-00500-s001.zip › Supplementary_Material_S7.pdf]

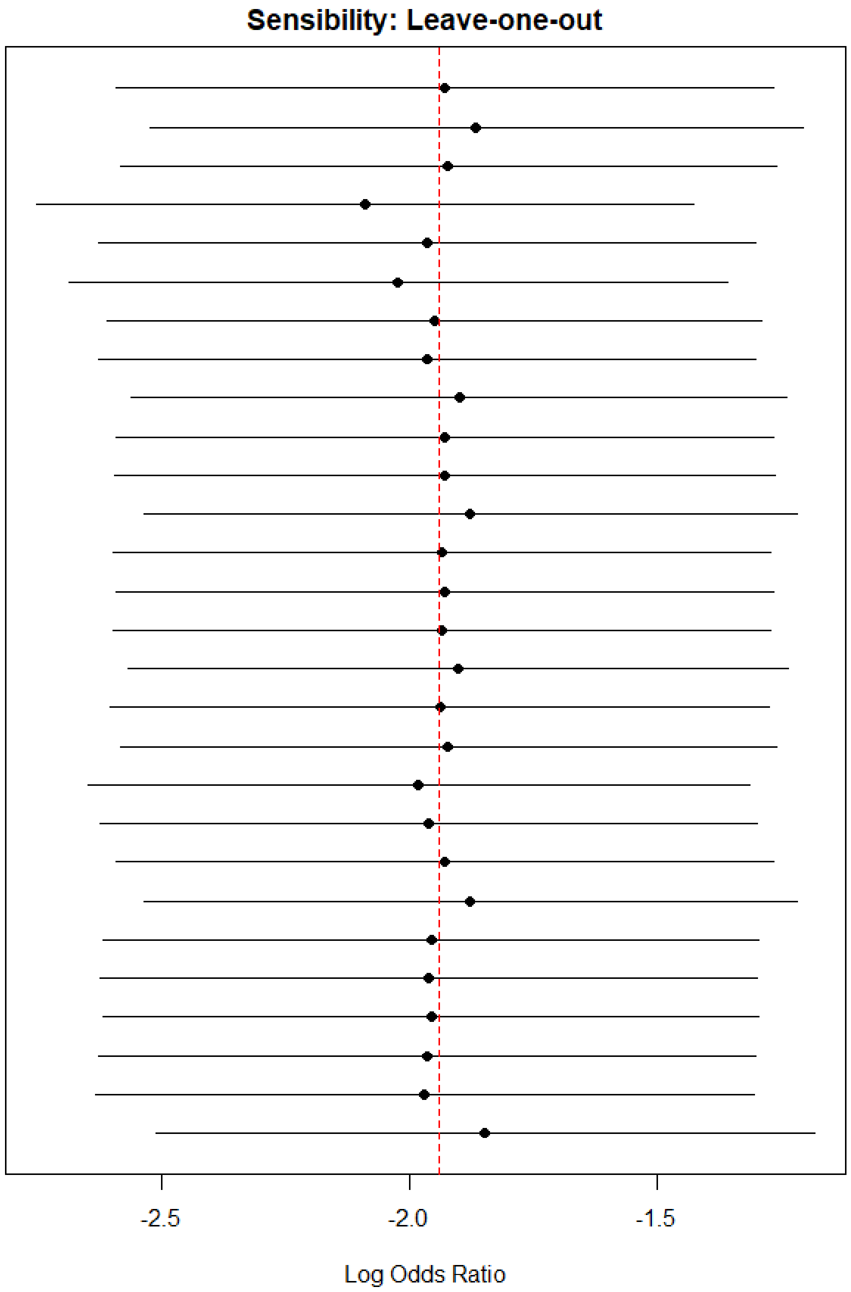

Figure S1. Forest plot of the sensitivity analysis (leave-one-out). Each point represents the pooled log Odds Ratio recalculated after sequentially removing one study at a time. Horizontal lines indicate the corresponding 95% confidence intervals. The vertical dashed line represents the overall pooled effect estimate from the full random-effects model (REML). The stability of the pooled estimates across iterations indicates the robustness of the meta-analytic results.

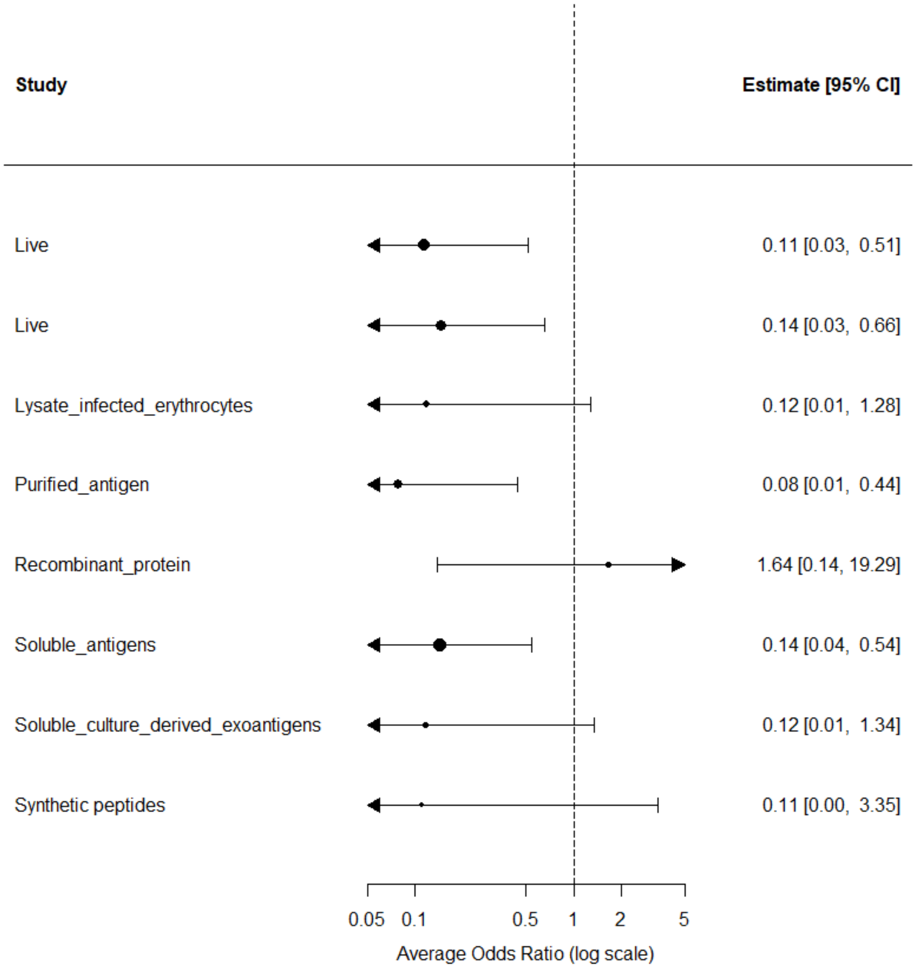

Figure S2. Forest plot showing the comparison of mortality between vaccinated and control groups by vaccine type.

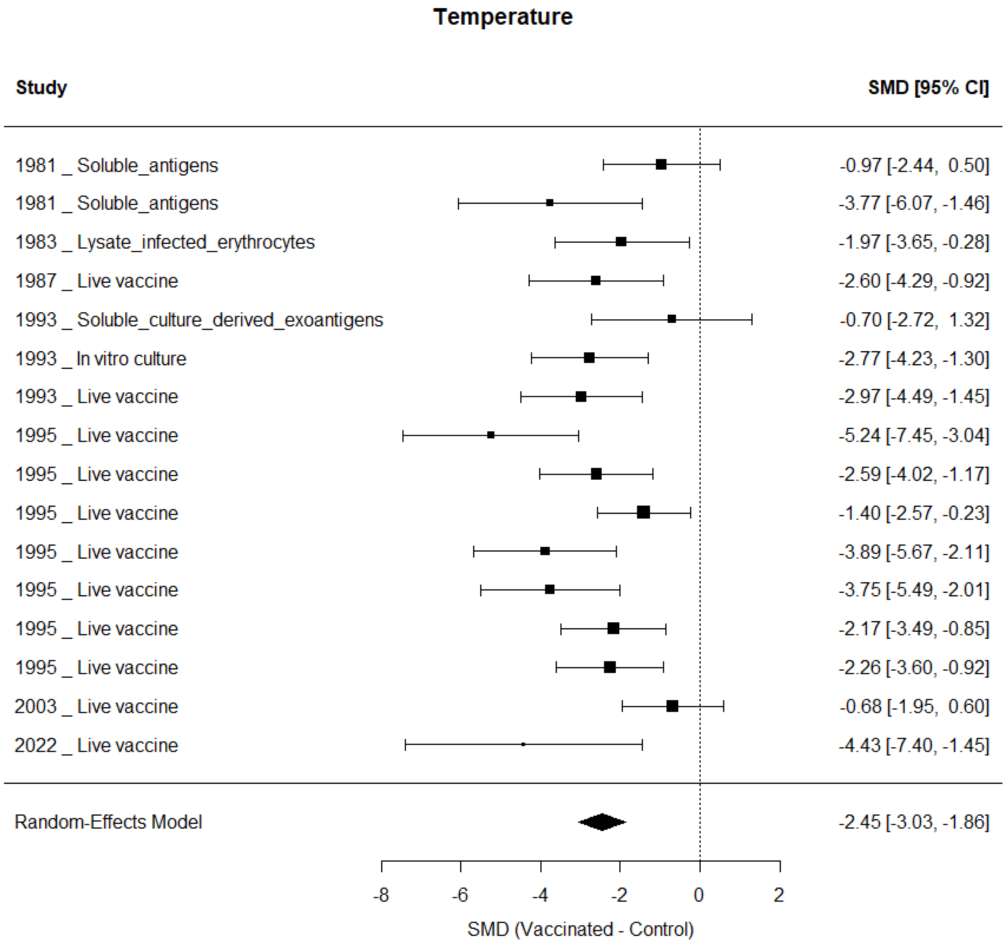

Figure S3. Temperature Controlled experiment. Forest plot showing the comparison between experimental and control groups. Individual studies are identified by year and intervention type. Black squares represent the effect size of each study, with size proportional to study weight, and horizontal lines indicate 95% confidence intervals. The vertical dashed line represents the null effect. The black diamond corresponds to the pooled estimate obtained under a random-effects model (REML), with its width representing the 95% confidence interval. Detailed quantitative results are described in the main text.

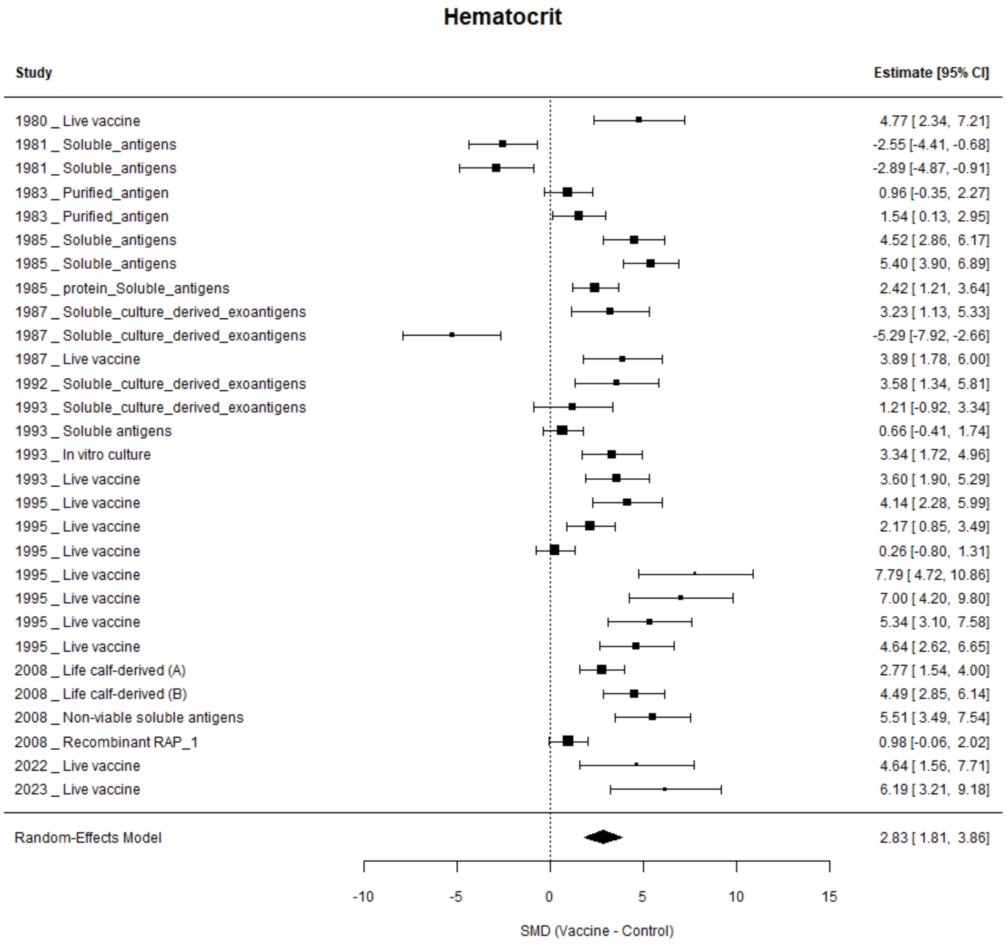

Figure S4. Hematocrit Controlled experiment. Forest plot showing the comparison between experimental and control groups. Individual studies are identified by year and intervention type. Black squares represent the effect size of each study, with size proportional to study weight, and horizontal lines indicate 95% confidence intervals. The vertical dashed line represents the null effect. The black diamond corresponds to the pooled estimate obtained under a random-effects model (REML), with its width representing the 95% confidence interval. Detailed quantitative results are described in the main text.

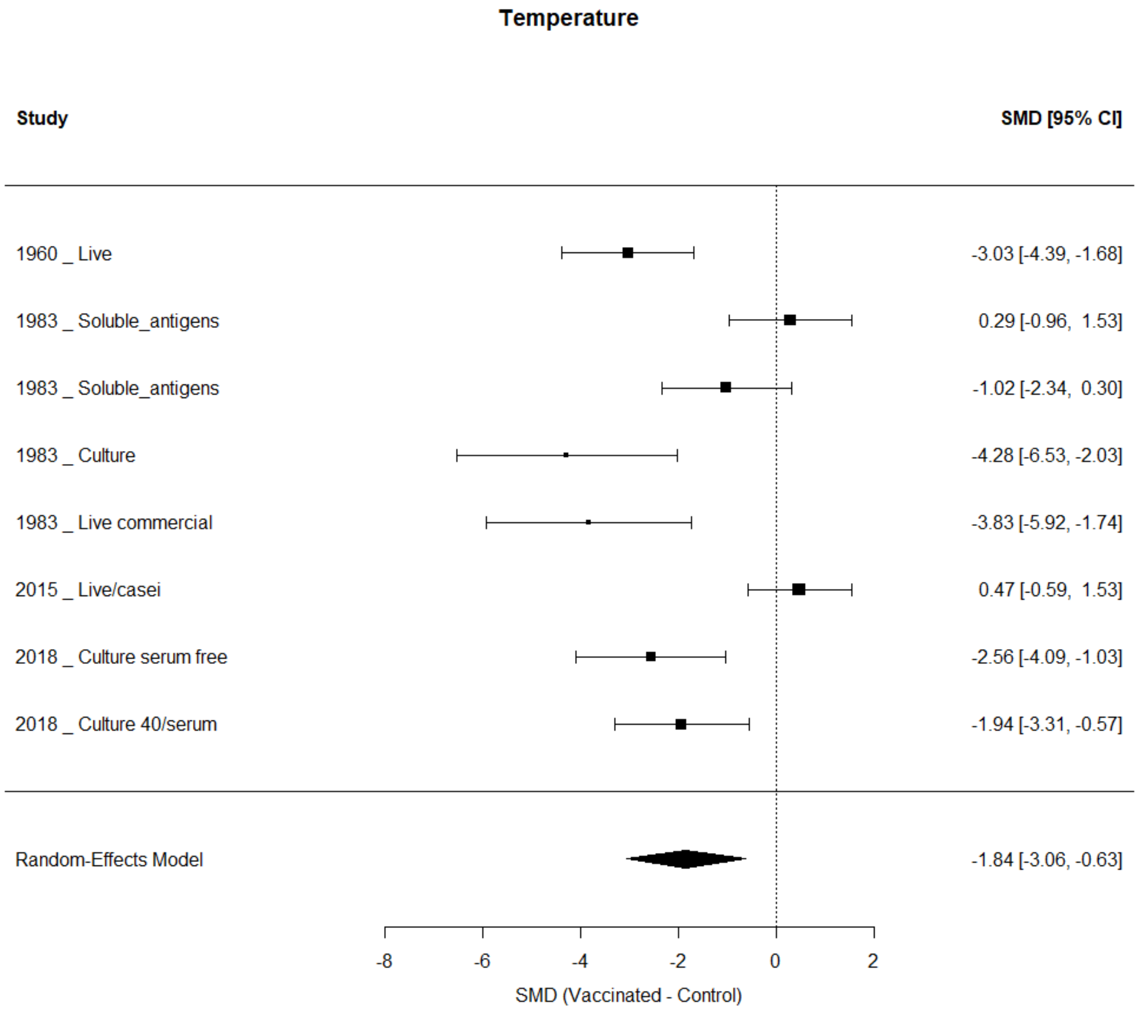

Figure S5. Temperature Field experiment. Forest plot showing the comparison between experimental and control groups. Individual studies are identified by year and intervention type. Black squares represent the effect size of each study, with size proportional to study weight, and horizontal lines indicate 95% confidence intervals. The vertical dashed line represents the null effect. The black diamond corresponds to the pooled estimate obtained under a random-effects model (REML), with its width representing the 95% confidence interval. Detailed quantitative results are described in the main text.

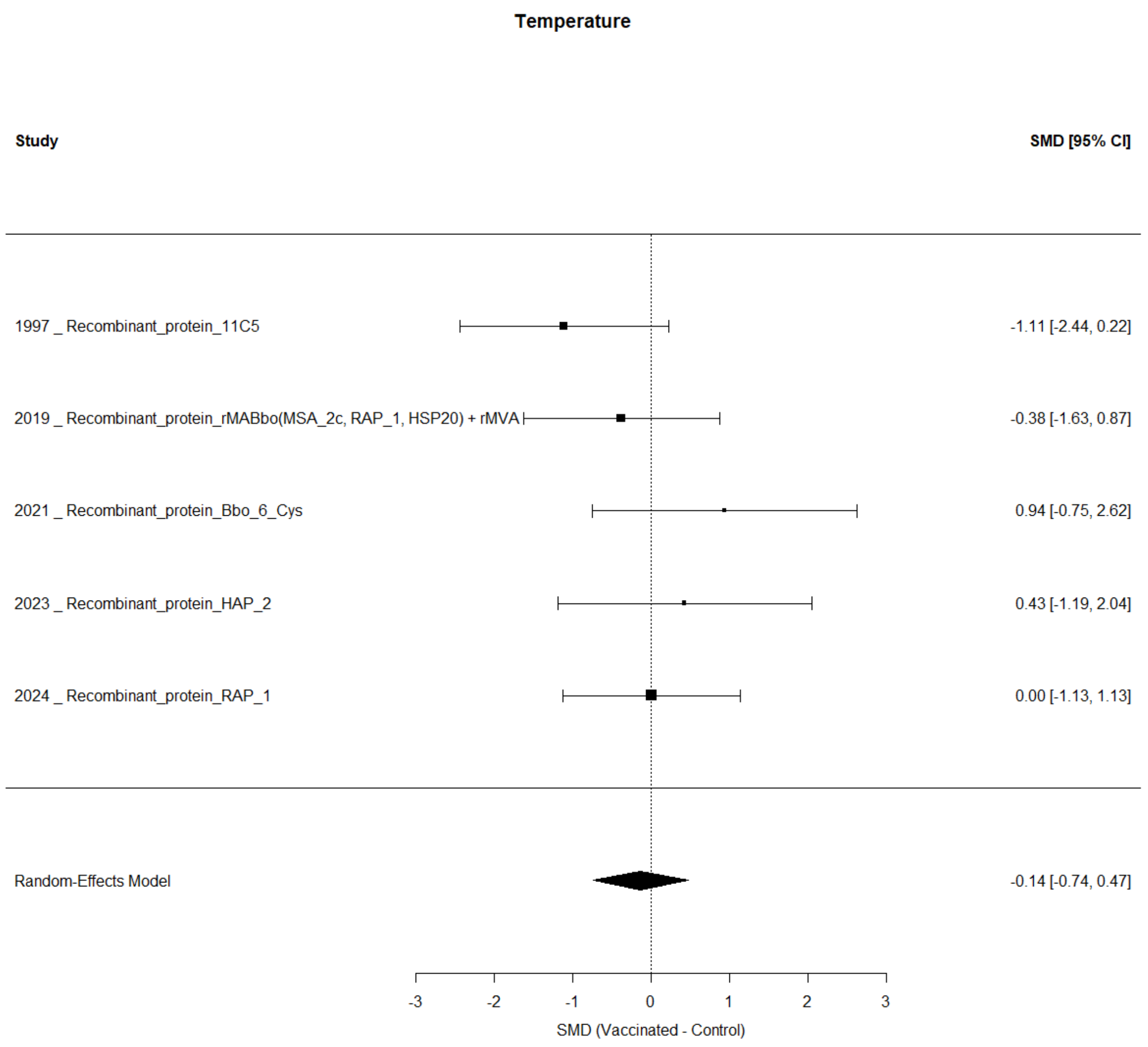

Figure S6. Temperature subunit vaccine controlled experiment. Forest plot showing the comparison between experimental and control groups. Individual studies are identified by year and intervention type. Black squares represent the effect size of each study, with size proportional to study weight, and horizontal lines indicate 95% confidence intervals. The vertical dashed line represents the null effect. The black diamond corresponds to the pooled estimate obtained under a random-effects model (REML), with its width representing the 95% confidence interval. Detailed quantitative results are described in the main text.

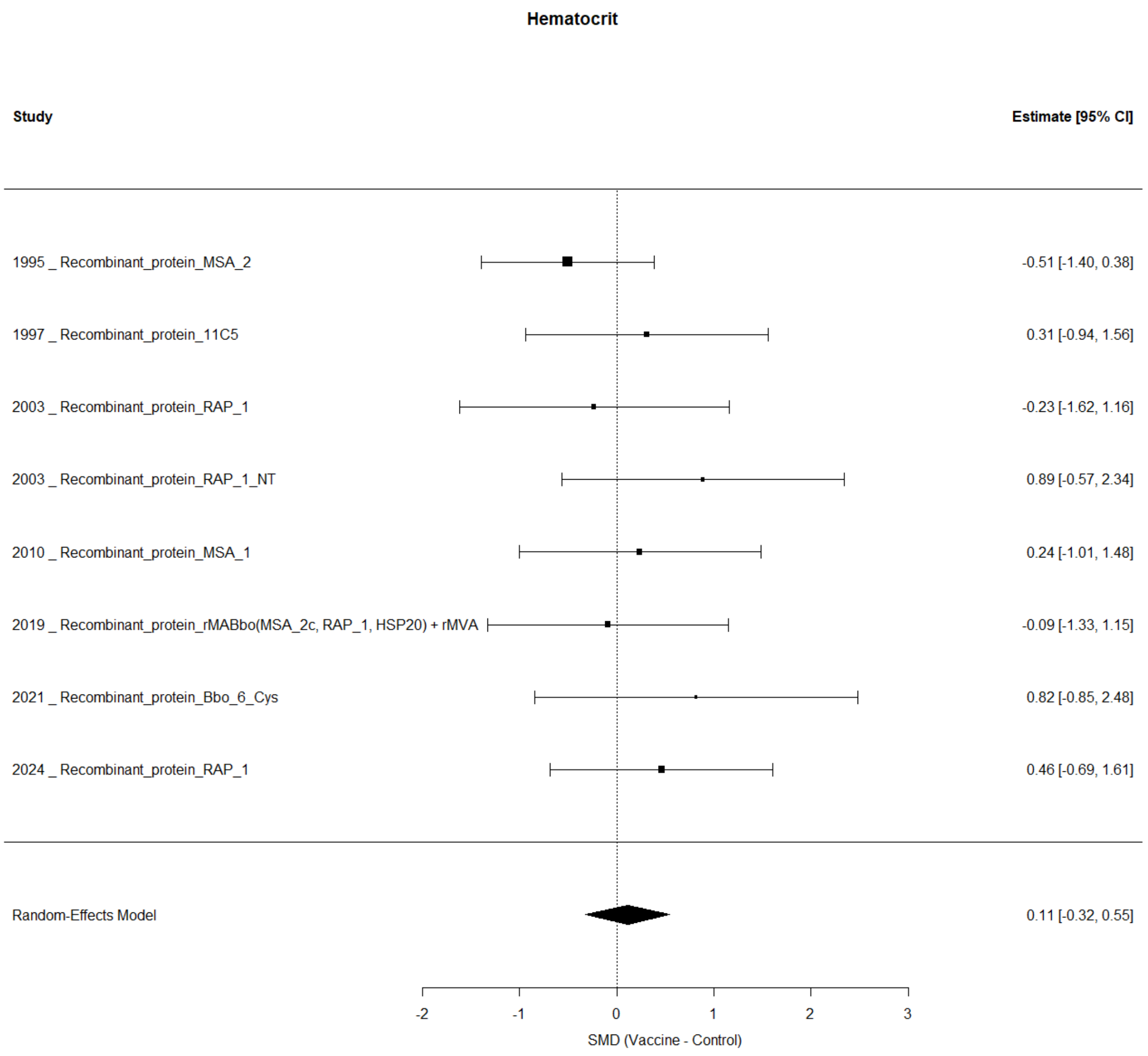

Figure S7. Hematocrit subunit vaccine controlled experiment. Forest plot showing the comparison between experimental and control groups. Individual studies are identified by year and intervention type. Black squares represent the effect size of each study, with size proportional to study weight, and horizontal lines indicate 95% confidence intervals. The vertical dashed line represents the null effect. The black diamond corresponds to the pooled estimate obtained under a random-effects model (REML), with its width representing the 95% confidence interval. Detailed quantitative results are described in the main text.

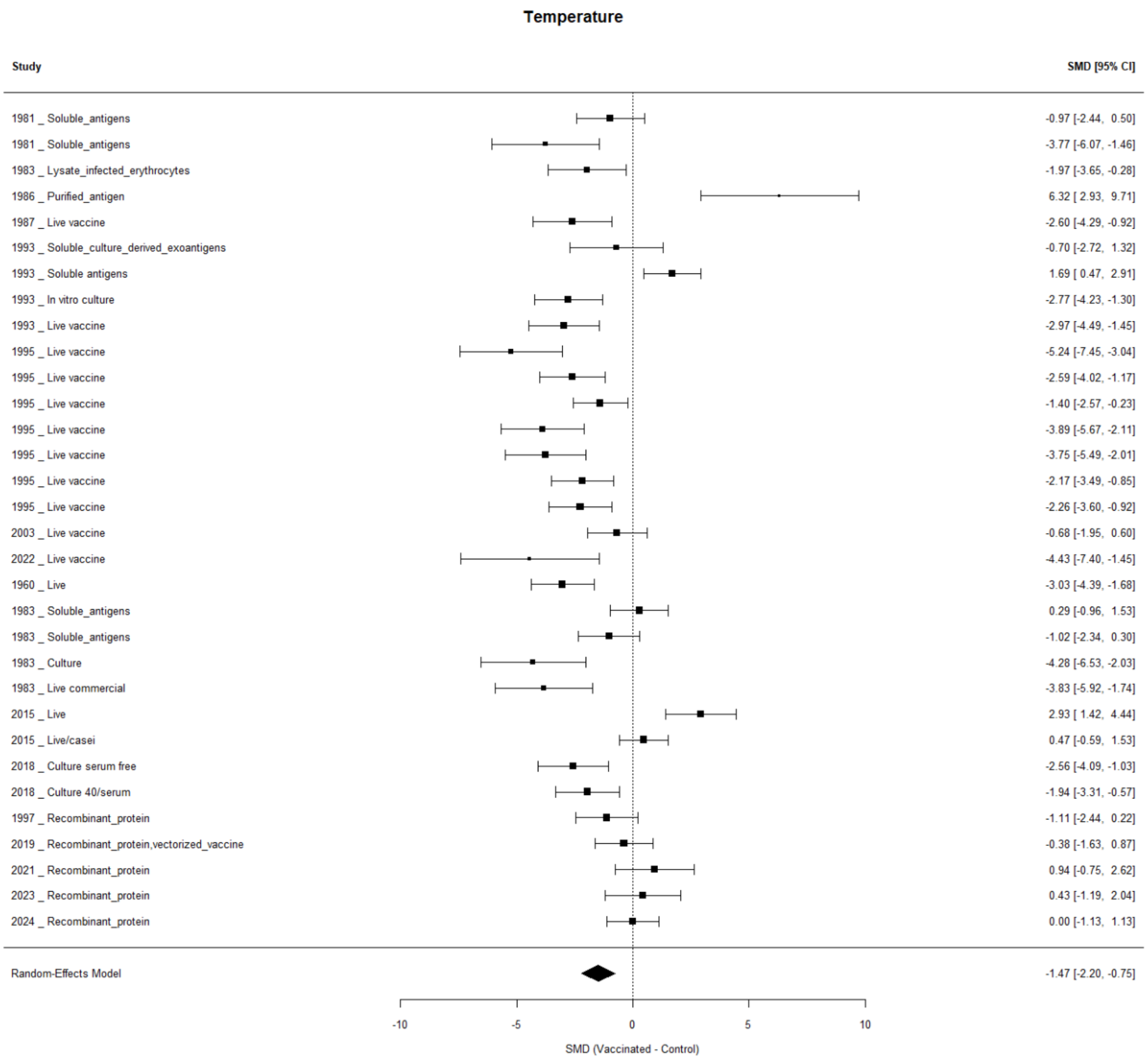

Figure S8. Temperature global experiments. Forest plot showing the comparison between experimental and control groups. Individual studies are identified by year and intervention type. Black squares represent the effect size of each study, with size proportional to study weight, and horizontal lines indicate 95% confidence intervals. The vertical dashed line represents the null effect. The black diamond corresponds to the pooled estimate obtained under a random-effects model (REML), with its width representing the 95% confidence interval. Detailed quantitative results are described in the main text.

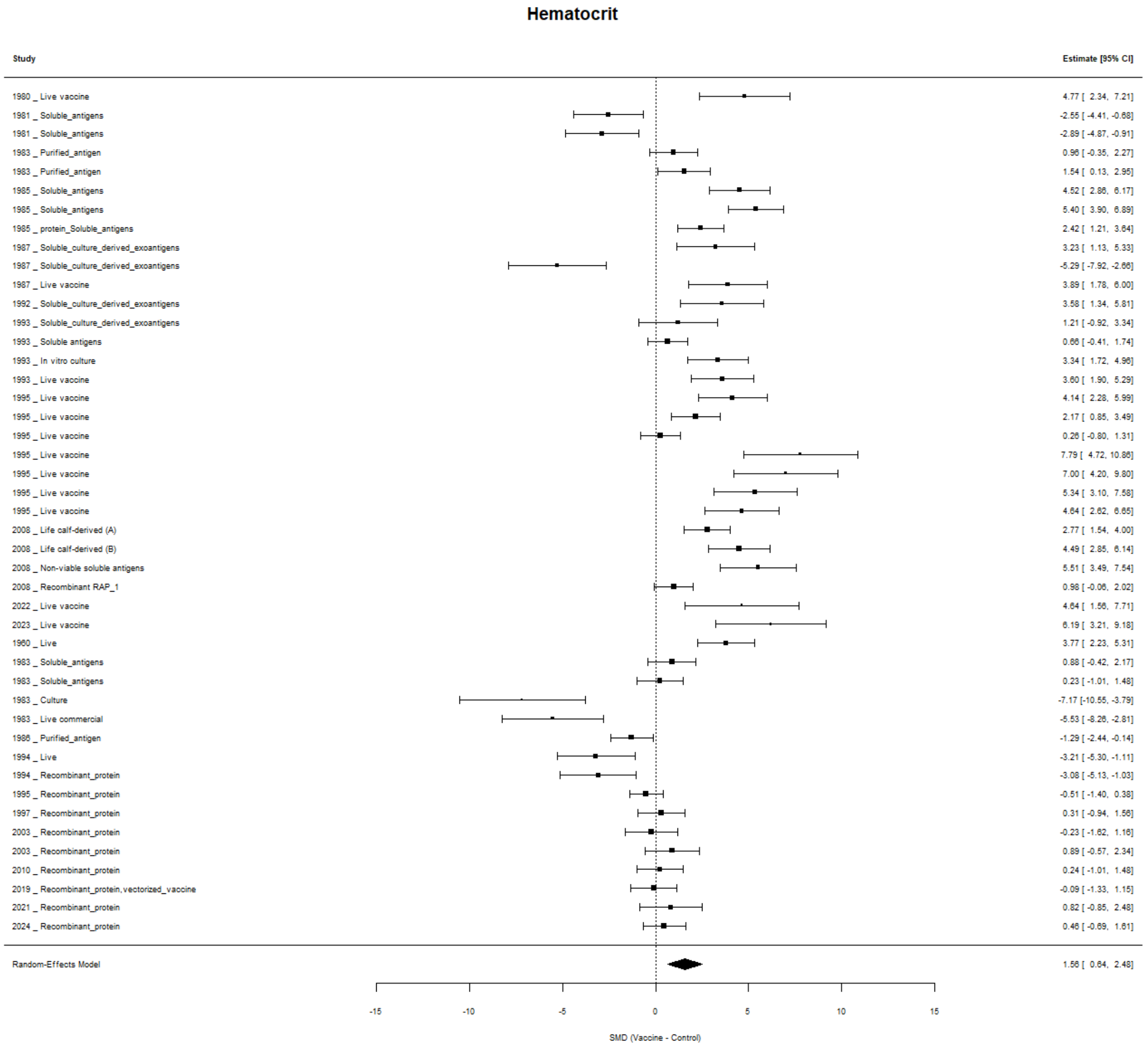

Figure S9. Hematocrit global experiments. Forest plot showing the comparison between experimental and control groups. Individual studies are identified by year and intervention type. Black squares represent the effect size of each study, with size proportional to study weight, and horizontal lines indicate 95% confidence intervals. The vertical dashed line represents the null effect. The black diamond corresponds to the pooled estimate obtained under a random-effects model (REML), with its width representing the 95% confidence interval. Detailed quantitative results are described in the main text.

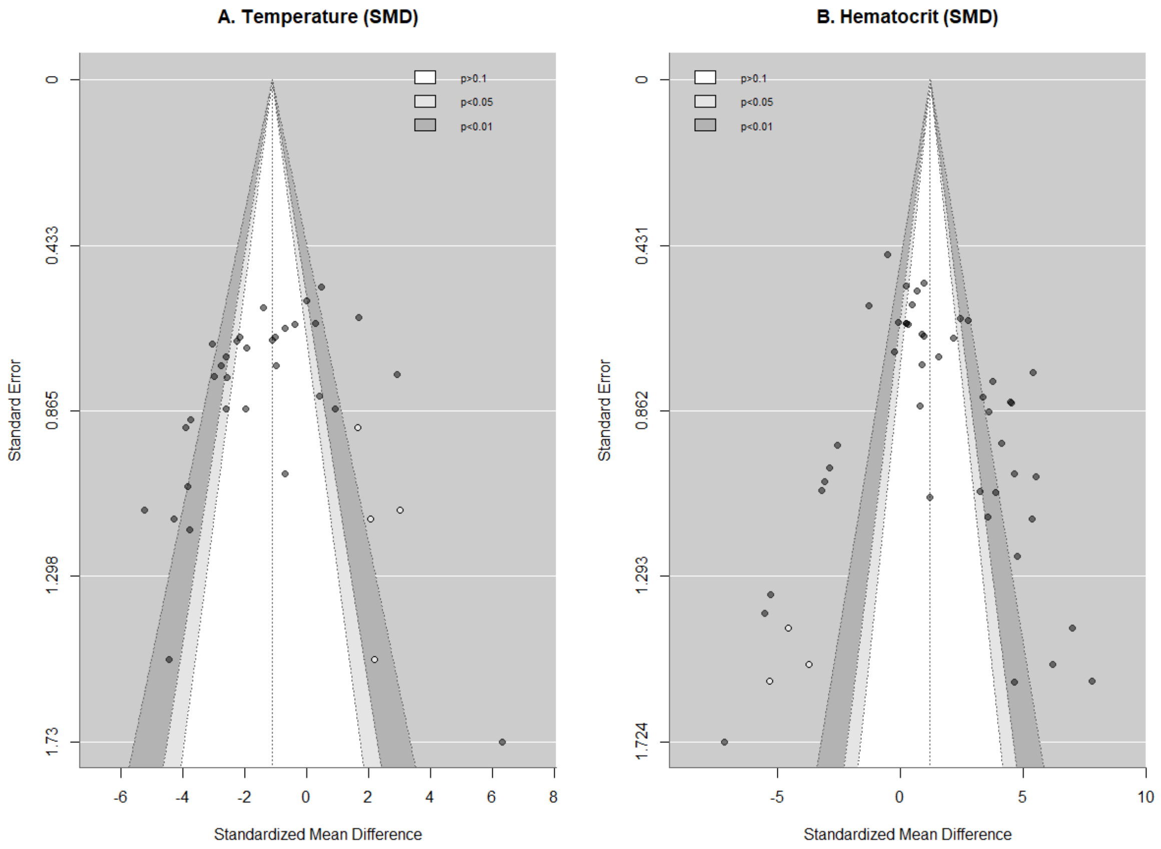

Figure. S10 Contour-enhanced funnel plots are shown for (A) temperature and (B) hematocrit outcomes (SMD). Shaded areas represent statistical significance regions. Open circles indicate studies imputed using the trim-and-fill method. Egger's test showed no evidence of significant asymmetry for either temperature ($p = 0.458$) or hematocrit ($p = 0.536$). However, Begg's test detected significant asymmetry for temperature ($p = 0.0025$), whereas no statistically significant asymmetry was observed for hematocrit ($p = 0.0642$).
