# Supplementary figures and images for: Six Decades of Global Research on Bovine Babesiosis Vaccines: A Comprehensive Systematic Review and Meta-Analysis
Source: Pathogens. 2026 May 6;15(5):500. doi: 10.3390/pathogens15050500 (PMC13209936; doi:10.3390/pathogens15050500)

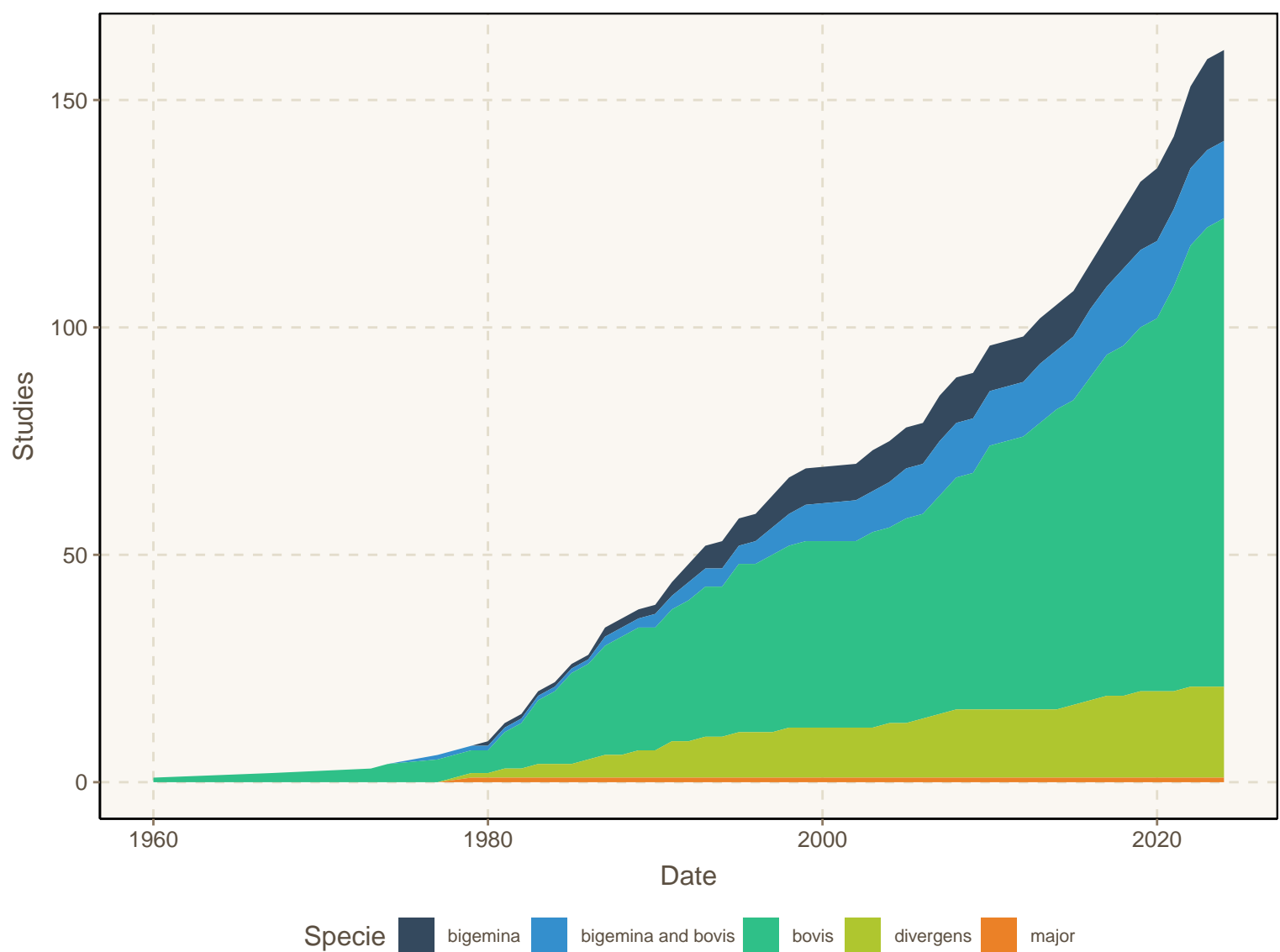

Supplement: Supplementary file 1 [file pathogens-15-00500-s001.zip › Supplementary_Material_S3.pdf]

# Number of publications per country

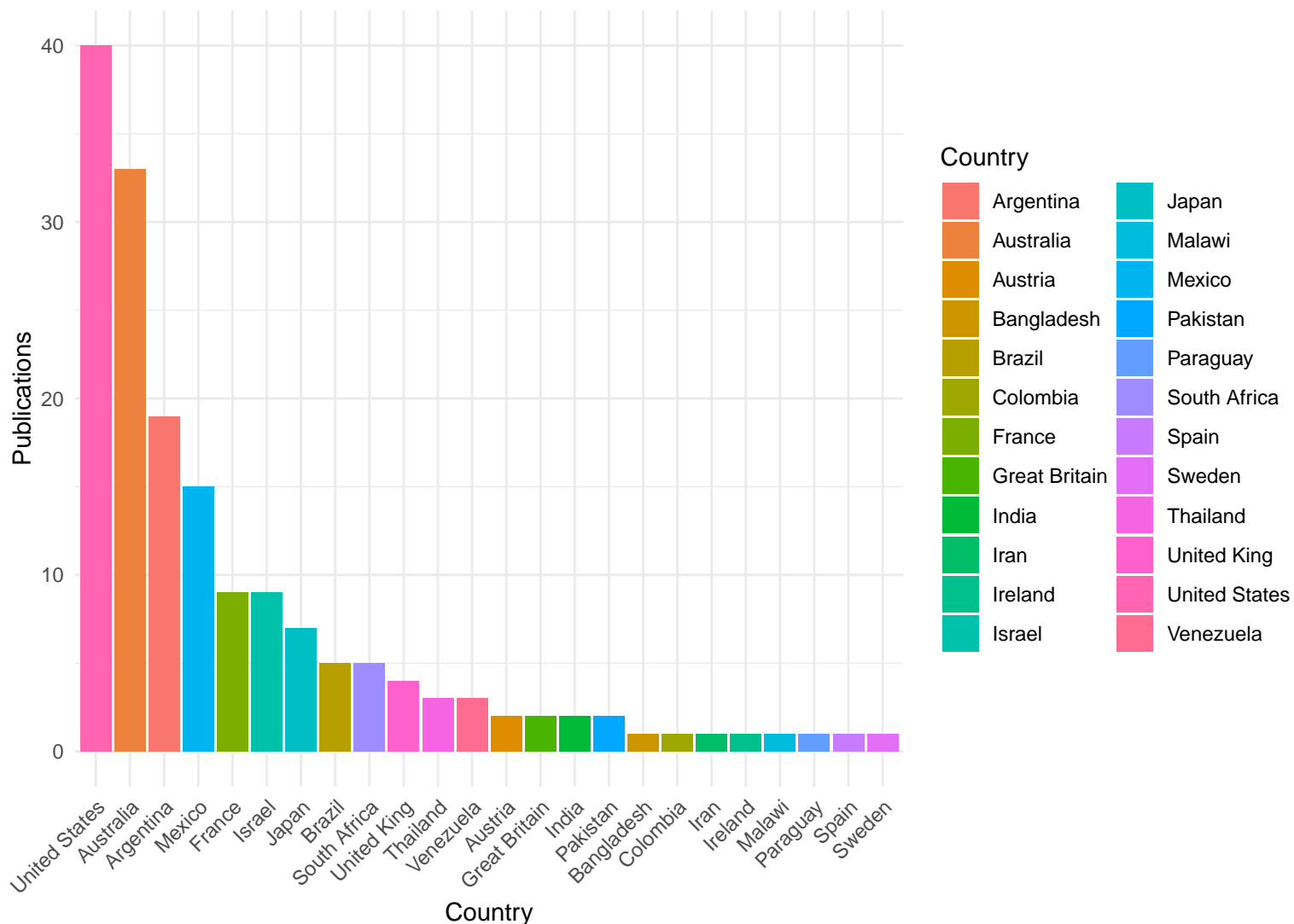

Supplement: Supplementary file 1 [file pathogens-15-00500-s001.zip › Supplementary_Material_S4.pdf]
